# Nanomedicines to Deliver mRNA: State of the Art and Future Perspectives

**DOI:** 10.3390/nano10020364

**Published:** 2020-02-20

**Authors:** Itziar Gómez-Aguado, Julen Rodríguez-Castejón, Mónica Vicente-Pascual, Alicia Rodríguez-Gascón, María Ángeles Solinís, Ana del Pozo-Rodríguez

**Affiliations:** Pharmacokinetics, Nanotechnology & Gene Therapy Group (PharmaNanoGene), Faculty of Pharmacy, Centro de investigación Lascaray ikergunea, University of the Basque Country UPV/EHU, Paseo de la Universidad 7, 01006 Vitoria-Gasteiz, Spain; itziar.gomez@ehu.eus (I.G.-A.); julen.rodriguez@ehu.eus (J.R.-C.); monica.vicente@ehu.eus (M.V.-P.); alicia.rodriguez@ehu.eus (A.R.-G.)

**Keywords:** in vitro transcribed messenger RNA (IVT mRNA), gene therapy, nanomedicine, immunotherapy, infectious disease vaccines, cancer immunotherapy, Chimeric Antigen Receptor (CAR) T cells, dendritic cells, protein replacement, gene editing

## Abstract

The use of messenger RNA (mRNA) in gene therapy is increasing in recent years, due to its unique features compared to plasmid DNA: Transient expression, no need to enter into the nucleus and no risk of insertional mutagenesis. Nevertheless, the clinical application of mRNA as a therapeutic tool is limited by its instability and ability to activate immune responses; hence, mRNA chemical modifications together with the design of suitable vehicles result essential. This manuscript includes a revision of the strategies employed to enhance in vitro transcribed (IVT) mRNA functionality and efficacy, including the optimization of its stability and translational efficiency, as well as the regulation of its immunostimulatory properties. An overview of the nanosystems designed to protect the mRNA and to overcome the intra and extracellular barriers for successful delivery is also included. Finally, the present and future applications of mRNA nanomedicines for immunization against infectious diseases and cancer, protein replacement, gene editing, and regenerative medicine are highlighted.

## 1. Introduction

According to the European Medicines Agency (EMA), a gene therapy medicinal product generally consists of a vector or delivery formulation/system containing a genetic construct engineered to express a specific transgene (‘therapeutic sequence’) for the regulation, repair, replacement, addition or deletion of a genetic sequence [1]. Nevertheless, a broader perspective is usually accepted from a scientific point of view, and the concept of gene therapy includes the therapeutic application of products containing any nucleic acid.

Gene therapy entered clinical trials in the early 1990s. Up to date around 17 nucleic acid-based products have been approved worldwide, and almost 2700 gene therapy-based clinical trials have been completed, are ongoing or have been approved for a broad range of applications. It is expected that nucleic acid-based products will have a substantial impact on the biopharmaceutical market in the near future [2]. Gene therapy has been clinically implemented primarily through two different approaches: Ex vivo or in vivo. In ex vivo therapy, cells are harvested from the patient or a donor, in vitro modified with the therapeutic nucleic acid and finally, re-infused into the patient. In vivo gene therapy is applied by direct administration of the vector containing the nucleic acid into the patient [3].

Depending on the final objective, gene therapy can be applied for gene augmentation, gene silencing, or gene editing [4]. Diverse nucleic acids are being used to address the development of these new medicinal products. DNA and messenger RNA (mRNA) induce protein expression, whereas, small interfering RNA (siRNA), microRNA, oligonucleotides or aptamers provide gene silencing [2]. Molecular scissor and gene editing approaches, such as zinc finger nucleases (ZFNs), transcription activator-like effector nucleases (TALENs), and clustered regularly interspaced short palindromic repeats (CRISPR)-associated nuclease Cas9 (CRISPR/Cas9) are also being developed.

The specific features of synthetic mRNA make it a promising alternative to DNA based products. Firstly, mRNA does not need the machinery of the nucleus to be functional, as DNA therapies do [5,6,7,8]. Therefore, once mRNA reaches the cytoplasm, translation of the encoding protein begins, being effective in both, mitotic and non-mitotic cells [9,10,11]. Secondly, mRNA has a better safety profile, because it does not integrate into the host genome; hence, the risk of carcinogenesis and mutagenesis usually associated with DNA is notably reduced [5,6,7,8,11]. In addition, it works without encoding additional genes, (i.e., those related to antibiotic resistance) [9]. Thirdly, the synthesis of the encoded protein is fast, and its expression is temporary [6,9]. The onset of expression is usually detected within 1h after transfection with a peak in expression 5–7 h later [12]. Finally, the production of in vitro transcribed mRNA (IVT mRNA) is easier than the manufacturing of DNA, and it can be standardized, maintaining its reproducibility [7].

**Figure 1:**
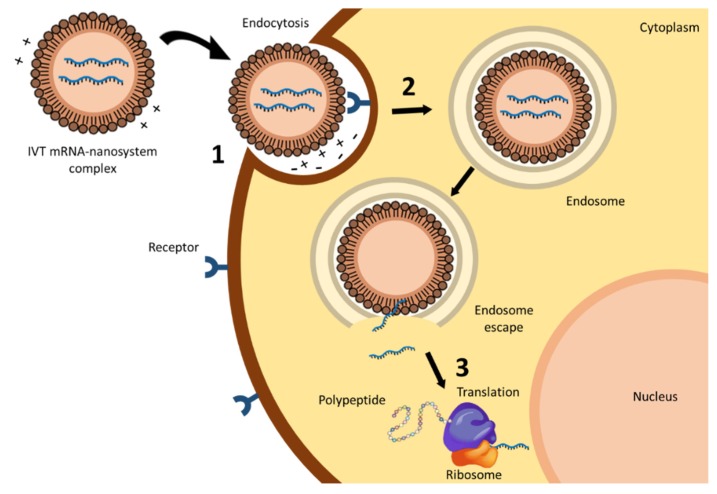
Intracellular barriers for in vitro transcribed (IVT) mRNA delivery: (1) Interaction between the delivery system and cell membrane, (2) endocytosis, and (3) endosomal escape and release of the mRNA to start the translation process.

Nevertheless, the use of IVT mRNA for clinical purposes has been mostly limited by its physical instability, its immunogenic capacity, and the difficulty in passing through the cellular membrane, due to the anionic nature of mRNA molecules [8,13,14]. The knowledge of mRNA structure has boosted modifications designed to improve stability and translation efficacy, and to reduce immunogenicity; however, optimized IVT mRNA still has to overcome several extracellular and intracellular barriers. As observed in Figure 1, IVT mRNA has to avoid degradation from extracellular degradative agents, such as ribonucleases, and interact with the target cell, cross the cytoplasmic membrane and diffuse in the cytoplasm to reach the ribosomes [7,15]. The formulation of IVT mRNA in suitable nanosystems or vectors is frequently a requirement for surpassing all these barriers. Non-viral vectors, and more specifically lipidic systems, are the most studied among delivery systems for IVT mRNA.

The first step for efficient internalization of IVT mRNA is the interaction between the delivery system and the cell membrane. The attachment to the cell surface may occur through electrostatic interactions between the system and the membrane surface, which is favoured for those systems presenting a cationic nature [16]. Cell binding can also be improved by incorporating ligands able to interact with specific cell surface receptors [17,18] into the vectors.

The main mechanism of cell entry is endocytosis. It comprises a variety of complex processes that determine the intracellular disposition of the mRNA. The vectors are included in endosomes by the invagination of the cell membrane. Endosomes mature and fuse with lysosomes, where the acidic environment and the presence of hydrolytic enzymes can degrade the vector and the nucleic acid. Therefore, endosomal escape before degradation is considered a bottleneck for successful mRNA therapy, and, as in the case of cellular internalization, the delivery system plays a crucial role. The foremost proposed mechanisms of endosomal escape include endosome disruption, active transport, or fusion of the delivery system with the endosomal membrane [19]. However, Patel et al. have recently identified that late endosome/lysosome formation is essential for the functional delivery of exogenously presented mRNA [20]. Therefore, for functional mRNA delivery, an appropriate biodistribution is necessary, but not enough; understanding cell-type specific endosomal escape mechanisms should help to design more appropriate strategies to optimize in vivo mRNA efficiency.

The present work reviews the strategies accomplished to optimize the functionality and efficacy of IVT mRNA. Besides the chemical modifications in its structure, the nanosystems and technological approaches developed for a successful IVT mRNA delivery will be described. Finally, the potential applications of mRNA nanomedicines will be discussed: Vaccination against infectious diseases, cancer immunotherapy, protein replacement, gene editing and regenerative medicine.

## 2. Structure of Synthetic IVT mRNA and Chemical Modifications

The production of IVT mRNA is usually carried out in cell-free systems, leading to easy standardization of clinical-grade manufacturing, which can be performed under Good Manufacturing Practices (GMPs). Fabrication costs of IVT mRNA under GMPs are substantially low as compared to recombinant proteins produced in eukaryotic cells [21]. It is important to select an efficient purification method of the IVT mRNA in order to eliminate aberrant (e.g., truncated) mRNA molecules, which are highly immunogenic contaminants and may lower translation efficiency [22].

Manufacturing of IVT mRNA by a cell-free in vitro transcription system requires a linearized DNA template which must contain a prokaryotic phage promoter sequence for the T3, T7, or SP6 RNA polymerases, the open reading frame (ORF) encoding the desired protein, the sequences corresponding to the regulatory untranslated regions (UTRs), and optionally, to a polyadenylated tail (poly(A) tail). [9,14,23]. When the poly(A) tail is not encoded directly in the DNA template, it can be added post-transcriptionally by enzymatic reactions with recombinant poly(A)polymerase of *E. coli* (E-PAP) [11,24]. Since the final IVT mRNA must be structurally similar to natural mRNA processed in the cytoplasm of eukaryotic cells, it also needs to be capped in 5’.

**Figure 2:**
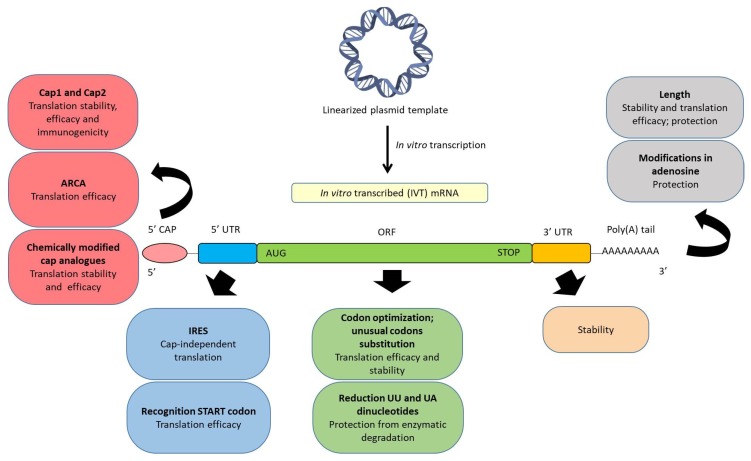
Representative scheme of the IVT mRNA structure and its principal modifications to improve the efficacy and the stability, and to reduce the immunogenicity.

A synthetic IVT mRNA consists of the following five fundamental structures, which can be chemically modified in order to optimize the translation process and the stability, and to regulate the immunogenicity: (a) Cap in 5’; (b) 5’ UTR; (c) an ORF, which has the starting codon AUG and the stop codon (UAA, UAG, UGA); (d) 3’ UTR; and (e) poly(A) tail. Figure 2 schematizes the main chemical modifications of these structural elements described up to date. It is known that chemical modifications influence in a specific manner the mRNA translation in different cell types. Therefore, addressing the precise intracellular behaviour of mRNA in the cell of interest will lead to further chemical modifications and extend the usefulness of mRNA as a biomedical product.

### 2.1. 5’ Cap

Eukaryotic native mRNA possesses a 5’ cap structure, known as cap0, formed by the union of inverted 7-methyl guanosine (m7G) to the first nucleotide of the mRNA by a 5’ to 5’ triphosphate bridge during the transcription process. This capping occurs by three consecutive enzymatic reactions, when the first 20–30 nucleotides of mRNA have been transcribed in the nucleus. 

Besides stabilizing the mRNA in the translation, splicing, polyadenylation and nuclear exportation processes, cap0 protects the mRNA from exonucleases. Additionally, cap0 interacts with cap binding proteins (CBPs), essentials for the nuclear export of mRNA, and also with the translation initiation factor 4E (eIF4E) in the cytoplasm, crucial for the initiation of translation [11,25,26]. Moreover, it could be used as an innate immune marker, which differentiates the viral RNA from cellular RNA [26].

In recent years, human enzymes that form two other types of 5′ cap, cap1 and cap2, have been identified [26,27]. In these cases, the m7G-specific 2′O methyltransferase (2′O MTase) methylates the second or third ribonucleotide at the 2′O or 3′O position of the riboses generating cap1 and cap2 structures, respectively. Cap1 and cap2 are less immunogenic than cap0, and they enhance translation efficiency [10]. Therefore, the introduction of cap1 or cap2 in the synthesis of IVT mRNA is a crucial factor in reducing the immunogenicity for applications in which immune response has to be minimized [24], but the incorporation of cap0 could be more useful in therapies in which the immune response is beneficial, such as vaccination.

In order to resemble the chemical structure of eukaryotic mRNA, synthetic mRNA transcripts can be capped after finishing the transcription (post-transcriptionally) or during the transcription (co-transcriptionally).

Post-transcriptional capping is usually carried out by using the enzyme machinery of recombinant *Vaccinia Virus* to perform the consecutive enzyme reactions to add the cap0, cap1 or cap2.

In co-transcriptional capping synthetic cap analogues are directly added during the transcription. This process is simpler than the enzymatic capping, but it also presents some limitations. On the one hand, all mRNA molecules obtained are not capped, due to the competition between the cap analogue and the guanosine triphosphate (GTP), which is the initiator nucleotide [14,26]. As a consequence, the uncapped mRNAs are immediately digested by nucleases and can induce an immune response. A strategy to reduce the stimulation of the immune system is the removal of the triphosphates of the 5′ end of uncapped IVT mRNA by using a phosphatase [9,28]. On the other hand, there is the risk of reverse orientation of the cap analogues, which impede the binding to the CBP and the posterior translation [11,24,29]. As an alternative, an anti-reverse cap analogue (ARCA) has been developed [11,24]. ARCA consists of a methyl group in the 3′-OH of the m7G nucleotide that enables the appropriate orientation of the cap and prevents the elongation of the mRNA along the wrong site [24,26]. Another traditional limitation of co-transcriptional capping is the inability to incorporate cap1 and cap2. However, recently, TriLink BioTechnologies has designed a new co-transcriptional capping technology, CleanCap^™^, able to incorporate cap1 or cap2 in the 94% of the mRNA molecules during the transcription process [30].

Finally, it also has to be taken into account that cytosolic decapping enzymes can remove mRNA cap. In order to provide resistance to the IVT mRNA against these enzymes, and therefore, extend its half-life, chemically modified cap analogues can be used. The resulting modified mRNAs can contain a phosphorothioate, phosphorothiolate, imidiphosphate and boranophosphate, among others [31,32,33,34,35,36].

### 2.2. ORF

The codon composition of the region that encodes the protein sequence, known as ORF, may also influence the translation efficiency and stability of the mRNA. The reduction of the quantity of UU and UA dinucleotides in the ORF has demonstrated to protect the IVT mRNA from decapping enzymes [37,38]. Additionally, since most amino acids are encoded by different synonymous codons, codon optimization strategies have been developed to improve the cost efficiency of recombinant protein production [39]. Codon optimization is mainly based on the substitution of multiple rare codons by other more frequent ones that encode the same amino acid. As a result, the rate and efficiency of the protein translation are increased [14,23,40]. However, the clinical application in humans is controversial, because in the last years it has been documented that synonymous mutations affect protein functions, and alterations in translation kinetics can lead to alterations in protein conformation [40,41,42,43].

### 2.3. Poly(A) Tail

The poly(A) tail in native eukaryotic mRNA is formed by 100–250 residues of adenosine [9,24,44]. It participates in the exportation process of mRNA molecules from the nucleus, interacts with initiation factors of the translation and prevents the degradation by nucleases through the interaction with poly(A)-binding protein, providing stability [45].

The poly(A) tail can be added to IVT mRNA directly during the transcription, if the DNA template encodes the poly(T) sequence, or post-transcriptionally by enzymatic reactions with recombinant E-PAP [11,24]. The addition during transcription is preferable, because the length of the poly(A) tail is more reproducible [14,23,24].

The length of the poly(A) tail influences the stability and translation efficiency of the IVT mRNA [46,47]; although a relatively long poly(A) tail seems to be appropriate, the optimal length may vary depending on the target cell. In HeLa (epithelial human) cells the increase of adenosine residues in the poly(A) tail from 14 to 98 improved the protein expression [48], whereas, in dendritic cells (DCs) a number of 120 adenosine residues enhances the translation efficiency, the protection and the stability of the IVT mRNA [49,50].

### 2.4. UTRs

The ORF is limited by the UTRs in both 5′ and 3′ sides. These non-coding regions do not participate directly in the codification of proteins, but their sequences, length and secondary structures are crucial for the regulation of the translation of the mRNA and the protein expression [51]. 5′ UTR is involved in the initiation of translation, which is considered the most intricate step among the whole process, whereas, 3′ UTR influences the mRNA stability and the extent of protein expression [52].

The presence of the internal ribosomal entry sites (IRES) in the 5’ UTR recruits the ribosome and induce a cap-independent translation initiation [53,54,55]. In order to improve translation efficiency, 5′ UTRs containing IRES from viral origin may be incorporated into IVT mRNA [37]. In these cases, translation is not dependent on eIF4E, as it is with cap0, and IVT mRNA expression is extended to cells where eIF4E levels are low [14]. However, the 5′ cap is still necessary for the protection of the mRNA against nucleases, and therefore, most IVT mRNAs contain both 5′ cap and IRES in their structure.

The Kozak consensus sequence, located in the 5′ UTR, also plays a major role in the initiation of the translation process. The Kozak sequence, defined as RCCAUGG, where *R* is a purine (*A* or *G*) was considered the preferred sequence for translation initiation in eukaryotes [56]. In this sequence, some nucleotides are more important than others; particularly, the −3 and the +4 positions, relative to the adenine of the starting codon AUG. To facilitate the recognition of the starting codon AUG, *G* nucleotide must be in the position +4, and *A*/*G* nucleotides must be in the position −3 [57].

Regarding the 3′ UTR, the presence of specific sequences of α-globin and β-globin mRNAs in this region improves the stability of IVT mRNA and the duration of protein expression, respectively [58,59]. In addition, the length of the 3′ UTR sequence may be modified to regulate the protein localization. For instance, in the case of CD47 membrane protein, long 3′ UTR induces the protein expression on the cell surface, whereas, short 3′ UTR results in the localization of the protein in the endoplasmic reticulum [60].

### 2.5. Modified Nucleosides

The incorporation of modified nucleosides into mRNA is a common strategy to reduce its immunostimulatory activity. Exogenous IVT mRNA induces innate immune responses by interacting with pattern recognition receptors (PRRs), including Toll-like receptors (TLRs) and cytoplasmic RNA sensors, such as retinoic acid-inducible protein I (RIG-I) [61,62]. It is known that uridine residues activate TLR7, and GU- and AU-rich RNA strands activate TLR7 and TLR8 [63,64]. However, the incorporation of modified nucleosides into the transcript, (i.e., pseudouridine (*Ψ*), N1-methylpseudouridine (N1m*Ψ*), 5-methylcytidine (m5C), N6-methyladenosine (m6A), 5-methyluridine (m5U), or 2-thiouridine(s2U)), avoids the activation of TLRs [65,66,67,68,69]. Some of them, such as *Ψ* and s2U, also abolish RIG-I activation [63]. In addition, the presence of m6A in the 5’ UTR have been proposed as an alternative to IRES, in order to favour cap-independent translation [70].

## 3. mRNA Nanomedicines

A key challenge for the clinical application of nucleic acid medicinal products entails the availability of delivery systems specifically adapted to their features and purpose. A vehicle for mRNA therapy, in addition to protecting it and providing specificity to reach the target cell, must afford an adequate intracellular disposition of the nucleic acid that enables the translation process, and all of this, preventing the activation of the immune response [71,72]. 

Currently, about 70% of the clinical trials with nucleic acids use recombinant viruses as delivery systems, such as retroviruses, lentiviruses, adenoviruses and adeno-associated viruses, among others [73]. Viral systems are viruses genetically modified to prevent their replication in the host cell; they present high transfection capacity, but also oncogenic and immunogenic potential. Moreover, viral vectors present a limitation regarding the size of the nucleic acid they can transport [74]. Despite the major advances achieved in DNA delivery with viral vectors, they do not play an important role in the case of mRNA-based products [7,75,76,77,78]. Non-viral systems are safe, their production is simple, economical and reproducible compared to viral vectors, and the size of nucleic acid to be packed is not usually an obstacle. However, their transfection efficacy is still a limitation, although it has been improved by different strategies and the efforts are still ongoing [75,77,79].

Therapeutic application of mRNA without the help of a delivery system presents important drawbacks [66]. Naked mRNA has been mainly applied ex vivo by using physical methods, including electroporation, microinjection and gene gun; these methods are able to disrupt the cell membrane and facilitate the entry of mRNA into the cell [80]. Electroporation consists in generating pores in a cell membrane through electric pulses. This technique has shown efficient mRNA delivery for tumor antigen loading of DCs [81]; even in some studies, mRNA electroporation has been more efficient than DNA electroporation, with faster and more homogeneous protein expression in vivo [82]. The direct injection of mRNA into the target cell by the use of microneedles (namely, microinjection) [83,84], and gene gun, in which naked mRNA is pneumatically shot into the target cell, have also been used for mRNA transfection [85,86,87,88]. In in vivo applications, intravenously administered naked mRNA is rapidly degraded by ribonucleases and the innate immune system can be activated. In fact, the half-life of naked mRNA has been estimated <5 min after intravenous administration, with a marked decrease in serum levels to about 10% after 5 min and to 1% after 60 min [89]. In contrast, subcutaneous, intramuscular or intranodal injections have been useful for naked mRNA delivery, especially when the activation of the immune system and a small amount of protein are required, as it happens in vaccination [90]. When administered subcutaneously, naked mRNA resulted more efficient than mRNA encapsulated in nanoparticles; the protein expression apparent half-life was 18 h after subcutaneous administration at the base of the tail and persisted for at least six days. In addition, protein expression decreased exponentially, which may render mRNA kinetics predictable [91].

**Figure 3:**
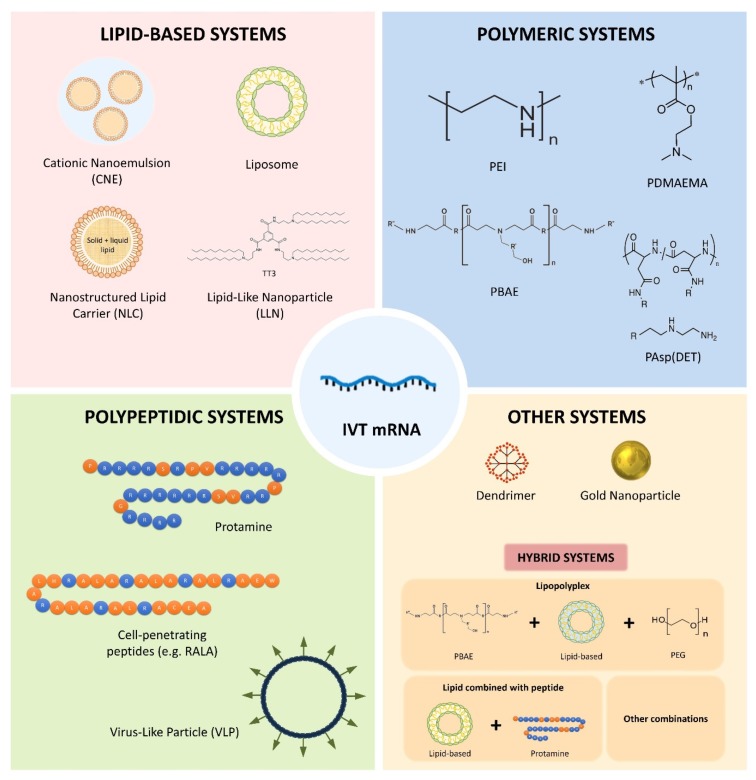
Representative scheme of chemical nanocarriers for mRNA delivery.

Chemical nanocarriers are nowadays at the forefront of pharmaceutical research for mRNA delivery. Thanks to the advances in material sciences, the rapid progress of nanotechnology, and in nucleic acid chemistry, extensive research is currently ongoing to develop new systems [81]. In this sense, a universal delivery system that can be used for any therapeutic application cannot be expected; mRNA carriers must be specifically designed to individual disease conditions. Chemical nanocarriers are formed by synthetic or natural biocompatible components that form complexes with the mRNA, and vary in composition, size, shape and physico-chemical characteristics. In addition to protecting the nucleic acid from degradation and denaturation, they all should facilitate the transfection process, being minimally toxic and avoiding immunological responses [92]. Moreover, these delivery systems could also be useful to programme the release profile of the active substance, improve the pharmacokinetic profile, reduce the toxicity to healthy organs/tissues and increase the blood circulation time [71,93]. As shown in Figure 3, nanocarriers for mRNA delivery evolve into various forms, including lipidic, polymeric and polypeptidic systems, dendrimers, gold nanoparticles, and hybrid systems. Considering the increased knowledge and current availability of tools to design novel nanomaterials, it is expected that new formulation strategies can further improve pharmacological profiles, and thus, expand mRNA utility.

### 3.1. Lipid-Based Systems

Lipid-based vectors are among the most widely used non-viral nucleic acids carriers. The main component of lipidic systems is cationic lipids, which are able to interact with the mRNA by electrostatic interactions, leading to the formation of a complex called lipoplex [78].

DOTMA (N-[1-(2,3-dioleyloxy)propyl]-N,N,N-trimethylammonium chloride) was the first synthetic cationic lipid utilized to complex IVT mRNA. The system containing the IVT mRNA luciferase delivered the nucleic acid in human, rat, mouse, xenopus (frog) and drosophila cells in vitro [94]. DOTAP (1,2-dioleoyl-3-trimethylammonium-propane) is another traditional synthetic lipid derived from DOTMA, which is more economical to synthesize and presents greater efficacy [78]. DOTAP has been frequently combined with the zwitterionic lipid DOPE (dioleoylphosphatidylethanolamine), to prepare colloidal systems able to bind the nucleic acids. This mixture facilitates the endosomal escape under acidic pH conditions, thanks to the capacity of DOPE to modify the lipoplex from a bilayer model to hexagonal phase II (*H*_II_) structures, known to induce supramolecular arrangements which result in the fusion of lipid bilayers [7,95]. The use of DOTAP alone or in combination with the helper lipid DOPE has been applied to mRNA delivery [96,97]. More recently, ionizable lipids with lower toxicity, but preserving the transfection capacity, such as 1,2-dioleoyl-3-dimethylammonium propane (DODAP) or 1,2-dioleyloxy-N,N-dimethyl-3-aminopropane (DODMA), have been developed as an alternative to conventional cationic lipids [5]. While cationic lipids present alkylated quaternary ammonium groups and retain their cationic nature regardless of the pH, ionizable lipids acquire positive charges, due to the protonation of free amines as pH decreases [13]. These new lipids are neutral at physiological pH but positively charged inside the endosome, when pH values are below its pKa. The electrostatic interactions between naturally occurring anionic lipids in endosomal membranes and the ionizable cationic lipids have been proposed as the underlying mechanism of nucleic acid release [88]. These interactions are able to promote membrane lytic nonbilayer structures, such as the hexagonal H_II_ phase, culminating in the intracellular mRNA delivery [98]. Nowadays, nanocarriers containing ionizable cationic lipids are among the leading delivery system candidates with promising applications for siRNA and mRNA delivery [78].

Cationic lipids can be formulated to prepare cationic nanoemulsions (CNEs) and lipid nanoparticles (LNPs) [75]. CNEs consist of a dispersion of an oil phase stabilized by an aqueous phase containing the cationic lipid. These nanoemulsions present a droplet size distribution of about 200 nm, and are mainly used to formulate mRNA vaccines [2,9]. For instance, a self-amplifying RNA (SAM) vaccine, expressing Human Immunodeficiency Virus (HIV) type 1 envelope, formulated in a CNE induced potent immune responses in rhesus macaques [99]. Brito et al. [100] developed a well-tolerated and immunogenic SAM vaccine based on CNEs. This SAM vaccine-elicited immune responses in a variety of animal models (including mice, rats, rabbits, and nonhuman primates) at much lower doses than those required for pDNA vaccines.

LNPs include liposomes and other lipid-based nanoparticles, and they are regarded as one of the most developed systems for mRNA delivery. Currently, several LNP platforms are at the forefront of clinical trials. Indeed, they are clinically validated delivery systems for RNA therapy. In the beginning, LNPs were considerably promising for the delivery of siRNA, being their utility as mRNA delivery agents more recent [101]. In this sense, Patisiran (ONPATTRO™), a siRNA formulated in LNPs targeted to inhibit hepatic transthyretin protein, received a recent FDA approval, meaning significant progress in the field [102]. This product contains a novel ionizable lipid, Dlin-MC3-DMA (MC3) [103], and after its success, several groups have managed to use MC3 as a vehicle for transferring mRNA [104]. The previous experience in siRNA formulation is benefiting the development of mRNA nanosystems, although mRNA has a different structure which may interfere with the packing capacity of nanoparticles [101]. For an optimal mRNA release, the delivery systems should contain less ionizable lipid and cholesterol and more phospholipid and polyethylene glycol (PEG) [5] than in the case of siRNA.

Liposome based formulations are amphiphilic spherical vesicles formed by one or more lipid bilayers enveloping an aqueous core with size ranging from 20 nm to a few microns. They generally contain a cationic lipid combined with: (a) A helper lipid that supports the bilayer structure and facilitates the endocytosis; (b) cholesterol to stabilize the lipid bilayer of the LNP; and (c) a PEG-lipid. PEG lends the nanoparticle a hydrating layer to improve colloidal stability, reduces protein adsorption and non-specific uptake, and prevents reticuloendothelial clearance [13,90]. Additionally, helper lipids, such as DSPC (1,2-distearoyl-sn-glycero-3-phosphocholine), DOPE and POPE (1-palmitoyl-2-oleoyl-sn-glycero-3-phosphoethanolamine) are frequently used in these systems [44]. The in vivo delivery of mRNA by using this kind of lipid-based system was already evaluated in 1994 [105], demonstrating a comparable efficacy to liposome-DNA complexes in accomplishing in situ tumor transfection. That study also showed that mRNA might be considered as an alternative to pDNA for genetic immunopotentiation applications. More recently, LNPs containing an ionizable cationic lipid, phosphatidylcholine, cholesterol and PEG-lipid were used to encapsulate nucleoside-modified mRNA encoding the pre-membrane and envelope glycoproteins of a strain from Zika virus [106]. A single intradermal low-dose immunization elicited potent and durable neutralizing antibody responses and protective immunity in mice and non-human primates. SAM vaccine platform is another example of a synthetic mRNA delivered by LNPs. SAM vaccine encoding an influenza H1HA antigen from N1H1 virus, and formulated with 1,2-dilinoleyloxy-3-dimethylaminopropane, 1,2-diastearoyl-sn-glycero-3-phosphocholine, cholesterol and PEG-DMG 2000, has demonstrated to be immunogenic in mice at low doses, and to elicit antibody responses that were comparable to the licensed vaccine [107]. The use of mRNA-liposomes in cancer therapy has increased dramatically, since the first study in 1999. Zhou et al. [108] used hemagglutinating virus of Japan (HVJ)-based liposomes for the synthesized mRNA encoding gp-100. Direct injection into the mouse spleen induced gp100 antibody expression and responses against B16 cells. Recently, the first-in-human, open label phase I study in ovarian cancer patients has been approved. Patients will be vaccinated intravenously prior, and during (neo)-adjuvant chemotherapy with a liposome formulated mRNA vaccine (W_ova1 vaccine) which includes three ovarian cancer- tumor-associated antigens (TAAs) RNAs (ClinicalTrials.gov Identifier: NCT04163094). CRISPR/Cas9 genome editing mediated by LNPs has recently provided in vivo evidence in animal models. Finn et al. [109] developed an LNP-mediated delivery method by using a biodegradable and ionizable lipid termed LP01 co-formulated with both Spy Cas9 mRNA and single guide RNA (sgRNA). A single administration enabled significant editing of the mouse transthyretin gene in the liver and resulted in >97% reduction in serum protein levels that persisted for at least 12 months.

A few marketed transfection reagents useful for mRNA transfection (for instance, MegaFectin^TM^, TransIT^TM^) are cationic liposome formulations based on DOTAP, DOPE and cholesterol [90]. As an example, expression was obtained after the intravenous injection to mice of MegaFectin^TM^ containing mRNA encoding red fluorescent protein [110]. Other cationic lipid-based transfection reagents commercialized have been widely used for mRNA delivery, such as Lipofectin^TM^, which is a mixture of DOTMA and DOPE, and Lipofectamine^TM^, which is a combination of DOSMA (the polycationic lipid 2,3-dioleyloxy-N-[2(sperminecarboxamido)ethyl]-N,N-dimethyl-1-propanaminium trifluoroacetate) and DOPE [90]. However, their use is restricted to in vitro studies, due to the toxicity associated with cationic charges, fast clearance, activation of immune response and low transfection efficacy in vivo [90,98].

Lipidoids, a new class of lipid-like delivery molecules comprising multiple hydrophilic groups and several lipid tails, were developed in 2008 as novel siRNA delivery agents [111]. Based on those results, more recently, a new class of lipid-like materials termed zwitterionic amino lipids (ZALs) have been applied for mRNA gene editing [112]. Intravenous co-delivery of Cas9 mRNA and targeted sgRNA from a single ZAL nanoparticle enabled CRISPR/Cas9 gene editing in mice.

In recent years, new lipid derivate systems, known as lipid-like nanoparticles (LLNs), have been developed for mRNA delivery. N1,N3,N5-tris(2-aminoethyl)benzene-1,3,5-tricarboxamide (TT) is formed by a phenyl ring, three amide linkers and three aminolipids chains [44]. Among them, TT3 was recognized as the principal compound in the optimized formulation, which increased in 350-fold the efficacy of luciferase-mRNA transfection [113]. More recently, TT3 LLNs delivered human factor IX (hFIX) mRNA and recovered the normal levels of the protein in IX-deficient mice [113]. TT3 LLNs were also optimized to deliver both Cas9 mRNA and sgRNA, demonstrating effective gene editing of hepatitis B virus DNA and the proprotein convertase subtilisin/kexin type 9 (pcsk9) gene [114].

Nanostructured lipid carriers (NLCs) are another type of LNPs used to deliver mRNA for vaccination. NLCs are colloidal structures composed by a core containing a mixture of solid and liquid lipids, resulting in an unstructured lipid matrix [115]. An important advantage of NLCs is their low toxicity respect to other lipid systems, as emulsions, which require high quantities of surfactants and cosurfactants. Additionally, production and sterilization of NLCs are easy and cheap compared to other systems. Specifically, sterilization of liposomal formulations is rather difficult, due to the sensitivity of phospholipids to heat and radiation [23], their production is costly, and batch-to batch reproducibility and large-scale manufacturing are difficult and expensive to achieve [116]. Better than for nucleic acid delivery, NLCs have been studied mainly for increasing the oral bioavailability of poorly soluble drugs [117]. Nevertheless, in a recent study [118], administration of replicating viral mRNA encoding Zika virus antigens formulated in NLCs, completely protected mice against a lethal Zika challenge; this achievement represents what might be the most potent approach to date of any Zika vaccine.

### 3.2. Polymeric Systems

The use of polymer nanoparticles has been intensively investigated for pDNA delivery, although few studies have addressed their use for mRNA. One key advantage of polymeric systems is the possibility of modifying their chemical properties to adapt them to the active substance. The binding of cationic polymers and nucleic acids leads to the formation of polyplexes [90]. Different cationic polymers have been studied for mRNA complexation, including polyethyleneimine (PEI), polyacrylates, poly(β-amino esters) (PBAEs) and poly(aspartamides) (PAsp), among others. However, despite the significant number of polymeric materials available, polymeric systems are not as clinically advanced as lipidic systems for mRNA-based therapies.

PEI was one of the first polymers used for nucleic acid delivery; it contains a large number of amine groups in its structure conferring it a positive charge. PEI may present both a linear and a branched conformation. Linear PEI contains secondary amino groups partially protonated at physiological pH, whereas, branched PEI contains primary and secondary groups, and a small number of tertiary amines. The presence of amino groups is responsible for the strong affinity to nucleic acids, including mRNA, and the cationic charges facilitate the interaction of the polyplexes with the cell membrane and the entry into the target cell. Moreover, amino groups allow ionization and confer high buffering ability. This buffering capacity, although under discussion, is responsibility of the “proton sponge effect” and enables the endosomal swelling and rupture by changing the osmolarity of acidic vesicles [71]. Nevertheless, the high density of positive charges is also related to in vivo toxicity by owing interactions with extra and intracellular proteins, destabilization of lipid cellular membranes and activation of the immune system [119,120]. New PEI derivatives have been designed to improve its biocompatibility and transfection efficiency. For example, jetPEI^®^, a linear PEI commercialized as an in vivo transfection reagent in mice, was firstly evaluated for pDNA and siRNA transfection, and later for mRNA delivery [121]. More recently, the administration of IVT mRNA with jetPEI^®^ by direct myocardial injection in the mouse demonstrated the expression of the protein in the lungs [122].

Polyacrylates have also been used for mRNA delivery, although with modifications in the side chain, needed to interact electrostatically with nucleic acids. One of the most studied polyacrylates is poly(2-dimethylaminoethyl methacrylate) (PDMAEMA), which presents lower affinity for mRNA than for pDNA; however, its PEGylation improves mRNA binding and transfection efficiency [123]. The development of triblock copolymers, formed by modifications in PDMAEMA structure, has demonstrated an improvement in transfection efficiency in both siRNA and mRNA systems. The modifications include: (1) The addition of amphiphilic materials, such as PEG methacrylate (PEGMA), to improve the stability and biocompatibility; (2) the incorporation of a hydrophobic butyl methacrylate segment (BMA) for fusogenicity; and (3) a pH-responsive diethylaminoethyl methacrylate (DEAEMA), to break the membrane of endosomes [71]. Triblock copolymer with PEGMA placed in the center of the copolymer chain showed high transfection efficiency and stability in macrophages and DCs, showing the potential of this system for mRNA-based vaccination approaches [124]. Oligoalkylamines grafted to an 8000 Da poly(acrylic acid) (PAA8k) scaffold complexed with chemically modified mRNA resulted in another kind of polyplexes with transfection capacity. Intravenous administration of PAA8k-luciferase mRNA systems in mice showed different luminescence signal in liver depending on their structure [125].

PBAEs are biodegradable and pH responsiveness copolymers synthesized by the addition of amines and acrylates via Michael-type reaction. The tertiary amine of its structure can electrostatically interact with the negative charge of nucleic acids. There is a wide variety of PBAEs delivery systems, due to their compatibility with other polymers, such as PEG, poly(lactic acid) (PLA), and poly(ε-caprolactone) (PCL) [126]. The use of PBAEs formulated with PEG-lipid improves serum stability of mRNA after intravenous administration [127]. Recently, inhaled delivery of IVT mRNA by hyperbranched PBAEs provided uniform distribution of luciferase mRNA in the lung, and protein expression lasted 24 h [128]. 

PAsp are synthesized by polymerization of DL-aspartic acid in orthophosporic acid medium and later addition of a nucleophilic amine [93]. The length of the amynoethylene side chain influences both the cationic charge and buffering capacity of each PAsp construct. For example, PAsp(DET), which is formed by the addition of 1,2-diaminoethane in the side chain to the PAsp, shows endosomal scape capabilities and biodegradability [90,128]. An odd-even effect of the amynoethylene repeated units has been described for different nucleic acid payload. PAsp containing even-numbered of amynoethylene units showed higher transfection with pDNA, whereas, an odd-numbered unit sustainably increased mRNA expression and enhanced resistance against [129,130]. PEG-PAsp polyplexes have displayed enhanced stability and lower cytotoxicity; for instance, IVT mRNA complexed with PEG-PAsp(DET) was intranasally administrated to mice for delivering brain-derived neurotrophic factor (BDNF), showing protein expression in nasal tissues for nearly two days [131]. PEG-PAsp(DET) also has been used to complex Bcl-2 IVT mRNA, being more effective than pDNA on reducing apoptosis in the liver of mice with fulminant hepatitis [132]. 

In addition to the most used polymers discussed above, it has been reported transfection of IVT mRNA with another kind of polyplexes. Nanoparticles formed by a core-shell structure of IVT mRNA complexed with the peptide protamine surrounded by PCL layers improved the stability of mRNA [133]. Chitosan, a biocompatible cationic glycopolymer containing amines, formulated as chitosan/hyaluronic acid nanoparticles, provided successful delivery of luciferase-encoding mRNA [134]. Self-immolative polycarbonate-block-poly(α-amino)esters, also known as charge-altering releasable transporters (CARTs), have demonstrated capacity to deliver mRNA thanks to their capacity to reduce chelative electrostatic anion-binding ability with the cationic polymer and facilitate endosomal escape [135,136]. CARTs are effective for in vivo delivery of mRNA with minimal toxicity in different cell lines and animal models via intramuscular, intratumoral, and intravenous administration. These CARTs have shown strong antigen-specific immune response against mRNA-encoded viral epitopes in a mouse model [137]. Another biodegradable ionizable amino polyesters (APEs) synthesized via ring opening polymerization of lactones with tertiary amino-alcohols are tissue-selective for mRNA delivery [138]. 

### 3.3. Polypeptidic Systems

Polypeptides consist of one or various amino acids disposed of in block or random sequences. They can provide biocompatibility and physicochemical properties to the delivery systems, thanks to the biodegradable naturally occurring monomeric units. Another advantage of nucleic acid delivery is the ability to adapt their cationic and endosomolytic properties, due to their structural flexibility [71].

Protamines are a family of small peptides with arginine-rich sequences obtained from fish sperm. Arginines confer positive charge, facilitating electrostatic interactions with the negative charge of the nucleic acid; in fact, protamine was described more than 50 years ago as an enhancer of RNA uptake [139]. The condensation of mRNA with protamine protects it against ribonuclease degradation, and the complex formed can activate TLRs acting as danger signals useful for vaccination [140]. Protamine-based formulations for IVT mRNA delivery are the second most used chemical systems in clinical trials, although far from lipidic systems. RNActive^®^ technology, developed by CureVac, is an mRNA vaccine platform based on protamine/mRNA complexes currently under clinical evaluation against rabies [138], and different cancers [141,142]. RNActive^®^ platform also has been tested preclinically against the influenza virus infection [143].

Cell-Penetrating Peptides (CPPs) have been used for nucleic acid delivery, due to their capacity to overcome cell membranes. Although the mechanisms of cellular internalization are not fully known, it is thought that CPPs may promote the grouping of negatively charged glycosaminoglycans of the cell surface, triggering macropinocytosis and lateral diffusion or directly disrupting the lipid bilayer [13]. A cationic CPP containing the arginine-rich amphipathic RALA motif was used as an mRNA vector for DCs. Nanocomplexes of RALA with *Ψ* and m5C modified IVT mRNA elicit potent cytolytic T cell responses against the antigenic mRNA payload [144].

Artificial viral coat proteins formulated as virus-like particles (VLPs) have been used as vehicles for transfection, due to their ability to assemble and protect mRNA. Li et al. [145] developed an mRNA vaccine as therapy for prostate cancer based on recombinant bacteriophage MS2 VLPs. This vaccine-induced strong humoral and cellular immune responses and protected mice against prostate cancer. In another work, artificial viral coat protein consisting of an oligolysine (K12), a silk protein-like midblock S10, and a long hydrophilic random coil block *C* was generated and complexed with mRNA to form rod-shaped VLPs. This system transfected cells with both EGFP and luciferase, but the efficacy was low compared to that obtained with a lipoplex transfection reagent [146]. More recently, VLPs prepared by fusing protein *G* of Vesicular stomatitis virus (VSV-*G*) with a ribosomal protein L7Ae of Archeoglobus fulgidus, resulted in the efficient delivery of EGFP in human induced pluripotent stem cells (iPSCs) and monocytes [147].

### 3.4. Dendrimers

Dendrimers are highly branched polymeric macromolecules with well-defined uniform sizes and shapes, and adaptable surface functionalities. Their basic structure encompasses a central core, repetitive branching units, and terminal groups [148]. Modified dendrimers, derived from polyamidoamine (PAMAM) have been extensively studied for nucleic acid delivery, due to their hydrophilic, biocompatible and non-immunogenic properties. Chahal et al. [149] developed a rapid-response and adjuvant-free vaccine based on a PAMAM dendrimer formulated in nanoparticles, wherein the antigens were encoded by encapsulated mRNA replicons. Intramuscular injection to mice of a single dose generated protective immunity against lethal Ebola, H1N1 influenza, and Toxoplasma gondii challenges. In a later study, this dendrimer-based nanoparticle was used to create a vaccine candidate that elicited Zika virus E protein-specific IgG responses. After immunization to mice, the authors detected a CD8+ T cell response against a unique H-2Db-restricted epitope [150].

### 3.5. Gold Nanoparticles

Gold nanoparticles (AuNPs) present features that make them an appropriate platform for nucleic acid delivery. AuNPs can be fabricated in a scalable fashion with low size dispersity, and they are easily functionalized by the formation of multifunctional monolayers and the inclusion of different moieties and targeting agents. Moreover, the in vivo toxicity and biodistribution, can be regulated by optimizing the particle size and surface functionality [151]. Yeom et al. [152] injected into mice xenograft tumors an IVT mRNA encoding Bcl-2-associated *X* (BAX) protein, a pro-apoptotic factor, loaded on AuNP-DNA conjugates. The mRNA released produced BAX protein, and tumor growth was inhibited.

### 3.6. Hybrid Systems

Hybrid systems are made up of the combination of various types of materials, including lipids, polymers and peptides, among others. Thereby, the hybrid system takes advantage of all the benefits of its individual components, offering greater functionality and flexibility [14,78], which may facilitate their clinical translation in the near future. However, the careful optimization required by a combination of such different components is an important becoming challenge in terms of scaling-up and clinical utility [71].

The combination of cationic lipids and peptides has been commonly studied for mRNA delivery. As an example, complexes formed by IVT mRNA, coding for the model antigen beta-galactosidase, condensed with protamine and encapsulated in liposomes provided in vivo protein expression, activation of cytotoxic T lymphocytes and production of IgG antibodies against the antigen [153]. In another study, the administration of lipid/protamine/IVT mRNA to mice bearing human lung NCI-H460 carcinoma, demonstrated better results in both efficacy and toxicity than the equivalent formulation with pDNA [154]. Lipofectamine^®^ with CRPPR-R9, a peptide containing nine arginine residues, efficiently transfected cultured mouse cardiac fibroblasts [155]. Cationic lipids have also been combined with inorganic nanoparticles. The transfection efficacy of mRNA-DOTAP, mRNA-apatite and mRNA-DOTAP-apatite was evaluated using an mRNA encoding the luciferase enzyme, being the mRNA-DOTAP-apatite complex the most effective [156].

Lipopolyplexes, the complexation of nucleic acids with cationic polymers and lipids, were among the first hybrids used for DNA and siRNA delivery, and later on for mRNA. Histidylated lipopolyplexes, synthesized by the combination of PEGylated derivative of histidylated polylysine and L-histidine-(*N*,*N*-di-n-hexadecylamine)ethylamide liposomes, incorporating a synthetic melanoma-associated antigen MART1 mRNA have been administered to mice as an mRNA cancer vaccine. The histidylated lipopolyplexes protected significantly injected mice against B16F10 melanoma tumor progression [157]. The subsequent mannosylation of the system targeted the mRNA into the DCs by the mannose receptor [158]. mRNA nanocomplexes formed with the polymer PLGA (poly(lactic-co-glycolic acid)), the cationic lipid-like compound (G0-C14) and a lipid-PEG were used to enhance the protein expression of the tumor-suppressor gene PTEN (phosphatase and tensin homolog deleted on chromosome ten). This hybrid system provided a high IVT mRNA PTEN transfection in prostate cancer cells, and led to significant inhibition of tumor growth when delivered systemically in multiple mouse models of prostate cancer [89]. A hybrid polymer-lipid nanoformulation for systemic delivery to the lung was prepared by co-formulation of PBAEs with lipid-PEG. This degradable polymer–lipid nanoparticle showed both enhanced serum stability and increased in vitro potency, delivering IVT mRNA in the lungs after intravenous administration in mice [127].

A nanomicelle-based platform was prepared by mixing IVT mRNA encoding an anti-angiogenic protein (sFlt-1), with PEG-polycation block copolymers. PAsp(TEP) was selected as the cationic segment of the block copolymer, and a cholesterol moiety was attached by hydrophobic interaction. PEG-PAsp(TEP)-cholesterol nanosystems produced efficient protein expression in tumor tissue, and remarkable inhibition of the tumor growth [159].

Another example of a multi-component delivery system is that formed with poly(glycoamidoamine) (PGAAs) brush nanoparticles. It has been used for intravenous administration of mRNA encoding erythropoietin (EPO) in mice [160]. First, three different PGAA polymers based on tartarate, galactarate, or glucarate sugars combined with three different amine-containing monomers were prepared. Polymer-brush materials were synthesized through ring opening reactions between PGAAs and epoxides, and incorporated into LNPs. Cholesterol, DSPC and mPEG2000-DMG (1,2-dimyristoyl-*sn*-glycero-3-phosphoethanolamine-*N*-[methoxy-(polyethylene glycol)-2000]) were added via a microfluidic-based mixing device to form the LNP polymer-brush nanoparticles.

Recently, DCs have been transfected with an mRNA delivery system combining both PLA-based micelles and the cationic CPP RALA. This hybrid nanoplatform offers the possibility of further multifunctionality by PLA core encapsulation [161].

## 4. Therapeutic Applications of mRNA

The growing knowledge of IVT mRNA design and manufacture, along with the advances in nanotechnology have conducted to broaden the potential therapeutic applications of mRNA-based medicines. According to preclinical and clinical trials, four major IVT mRNA applications can be considered: Immunotherapy (against infectious diseases and cancer), protein replacement, gene editing and regenerative medicine. Currently, all the clinical trials ongoing, both applying in vitro and in vivo strategies, are still in Phase I or II, with most of them focusing on immunotherapy, and especially on cancer therapy. Nevertheless, the successful transition of mRNAs from clinical studies to commercialization in the form of medicinal products requires still an important consideration related to the fabrication of large industrial batches: Optimization of IVT mRNA production and purification processes with further cost reduction [71]. 

### 4.1. Immunotherapy

The induction of an immune response by using mRNA has been the main application among mRNA-based therapies, with a number of mRNA vaccines being evaluated in clinical trials against infectious diseases and multiple types of cancer. Apart from these applications, a proof of concept for prevention of type 1 diabetes in mice, by administering modified T cells redirected against diabetogenic CD8+ T cells, has emerged as a new mRNA-immunotherapy application [162].

Additionally, passive immunization by mRNA encoding monoclonal antibodies is showing great biomedical interest. Given the rapidly growing market of therapeutic monoclonal antibodies, and the high cost of this type of medicines, the pharmaceutical industry is looking for alternatives approaches. mRNA is considered a good option, due to its simpler, faster and more cost-effective synthesis. Until now, pre-clinical studies in small rodents have demonstrated antibody titters from the first day after mRNA intravenous administration [163]. However, before moving on to the clinic, it is still necessary to highlight whether mRNA can lead to high antibody concentrations, often needed for a therapeutic effect.

Besides the general advantages of mRNA, previously mentioned, this active substance shows specific interesting features for immunotherapy: The immunostimulatory capacity, although it can also have potential toxicity, the transient nature of the antigen and the versatility of applications, including prophylaxis, therapy and personalized vaccines [164,165]. Several preclinical studies of mRNA vaccines are showing promising perspectives. However, the preclinical and human immunogenicity is not always the same, animal studies are not predictive of human efficacy, and there is a lack of knowledge about the targets and type of immune responses which are essential for effective therapy. These challenges highlight the need to carry out further studies on the correlation between the immune response mechanisms in animals and in humans, as well as of a better understanding of the diseases to be treated or prevented, for the clinical success of immunotherapy [14,165].

#### 4.1.1. mRNA Vaccines Against Infectious Diseases

**Figure 4:**
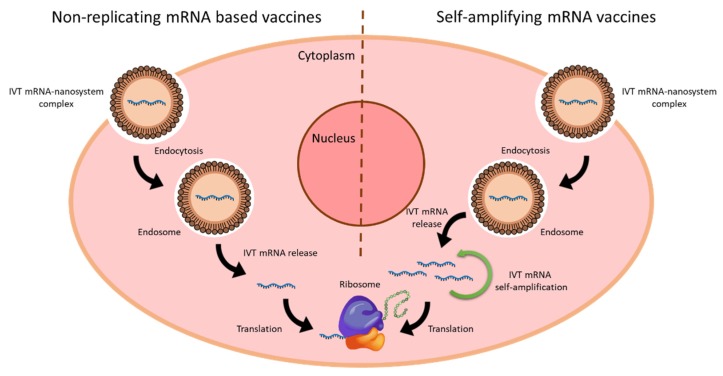
Representative scheme of the difference between non-replicating mRNA-based vaccines and self-amplifying mRNA (SAM) vaccines.

mRNA vaccines are emerging as potential substitutes of conventional vaccines, due to their advantages comparing with subunit, killed, live attenuated and inactivated pathogens containing vaccines, and with DNA-based vaccines [164]. Two kinds of mRNA-based vaccines, SAM and non-replicating vaccines, have been developed against infectious diseases. Figure 4 shows a representative scheme of the intracellular disposition of these two types of vaccines; in both cases the translation machinery of the cell is used to produce the specific antigen [13].

SAM vaccines, called replicon, contain a positive single-stranded RNA virus genome, especially from alphaviruses, where the genes encoding the replicative viral machinery are not modified, but structural genes are replaced by the antigen sequence. Therefore, SAM vaccines contain two different ORFs, one encoding proteins for RNA capping and replication, and another encoding the target antigen. SAM vaccines show an increased efficacy thanks to the ability of the replicon to self-replicate and amplify inside the cell, enabling a significantly higher antigen production for a given mRNA vector [14,165]. Another advantage is the capacity to encode different antigens in their sequence. This feature allows the implementation of different therapeutic strategies, such as a vaccine encoding both the antigen of interest and an immunomodulatory molecule to enhance the efficacy, a vaccine with both antigens of T and B cells to attack a wide variety of pathogens, or a vaccine against multi-subunit complex antigens [166]. However, among the limitations of the replicons are their large size (~10kb) and the inability to withstand many modifications of synthetic nucleotides and alterations of the sequences [13].

The first report of SAM vaccine was in 1994, when it was demonstrated that the IVT mRNA derived from the alphavirus Semliki Forest virus (SFV) activated the immune system against a heterologous antigen [167]. In later works, it was reported that a single low dose of naked SFV-derived IVT mRNA encoding respiratory syncytial virus fusion (RSV-F) protein, influenza virus hemagglutinin (HA), or louping ill virus prME produced antibody response and partial protection from lethal viral challenges in mice [168]. The development of LNPs have played an important role in the improvement of SAM vaccines efficiency. The administration of very low doses of LNP complexed with the RSV-F RNA replicon vaccine triggered a potent lymphocyte T and B responses in mice, and elicited a protective response against RSV in cotton mice [169].

As can be seen in Table 1, the use of SAM vaccines is under clinical evaluation for cytomegalovirus (CMV), influenza and HIV-1 infectious diseases. AVX601, a bivalent alphavirus replicon vaccine (alphavaccine) expressing three CMV proteins (gB, pp65 and IE1), was a vaccine candidate against CMV evaluated in healthy volunteers in 2007 (NCT00439803). The vaccine was well tolerated and induced neutralizing antibody and multifunctional T cell responses against the three CMV antigens [170]. The safety and immunogenicity of another alphavaccine (AVX502), expressing an influenza HA protein was evaluated in healthy volunteers of 18-40 years of age (NCT00440362) or 65 years of age or older (NCT00706732), respectively. The vaccine was safe and well tolerated in both trials, and, especially in the younger group, antibody and T cell responses were efficiently stimulated and persisted for the four-month study [171]. Finally, an alpha vaccine (AVX101) in which the alphavirus structural proteins were replaced with HIV-1 subtype C *gag* was administered subcutaneously in healthy HIV-1-uninfected adults for safety and immunogenicity evaluation (NCT00097838 and NCT00063778). Only low levels of binding antibodies and T cell responses were observed at the highest doses [172].

Non-replicating mRNA-based vaccines are engineered to resemble fully processed mature mRNA, and are formed by an IVT mRNA containing an ORF encoding the antigen of interest. The clinical trials currently ongoing for infectious diseases with non-replicating mRNA include prophylactic vaccines directed to rabies, Zika virus, CMV, human metapneumovirus, human parainfluenza, tuberculosis and influenza viruses (Table 1). All of them use an in vivo strategy by direct intradermal, intramuscular or subcutaneous injection of vaccines, with lipid nanosystems as the most used delivery system.

The first study of immunization by non-replicating mRNA vaccines against infectious diseases was reported in 2012 by Petsch et al. [143]. Synthesized mRNA encoding different influenza virus antigens based on CureVac’s RNActive^®^ platform were administered intradermally to mice, ferret and pigs. Later on, IVT mRNA encoding an optimized non-replicating rabies virus glycoprotein (RABV-G), also formulated as RNActive^®^ vaccine, was administered intradermally in mice and pigs [173]. The vaccine provided protection against lethal intracerebral challenge infection in mice, whereas, neutral antibodies were detected in pigs. The first clinical trial of this prophylactic mRNA-based vaccine (CV7201) in healthy adults demonstrated a reasonable tolerability profile (NCT02241135). The participants received three doses of CV7201 intradermally or intramuscularly by needle-syringe or needle-free devices. Vaccine-induced boostable functional antibodies against a viral antigen only when administered with a needle-free device [174]. Based on positive results from preclinical studies in both mice and nonhuman primates [138], a new intramuscular RABV-G mRNA vaccine encapsulated in LNPs (CV7202) is being tested in clinical studies (NCT03713086).

After successful preclinical results immunization against Zika virus by mRNA vaccination also has reached clinical evaluation [106,175]. The safety, tolerability and immunogenicity of two Zika vaccines containing mRNA encoding viral antigenic proteins formulated with LNP are under clinical evaluation in healthy adults (NCT03014089, NCT04064905).

The immunogenicity of vaccines based on LNP-complexed mRNA against influenza virus was also reported in mice, ferrets, and non-human primates [176]. Subsequently, the results from two clinical trials (NCT03076385, NCT03345043) have shown that the mRNA vaccines against H10N8 and H7N9 influenza viruses, formulated as LPNs, were well tolerated and elicited robust humoral immune responses in healthy adults [177].

Two clinical trials (NCT02413645, NCT02888756) evaluated mRNA vaccines as an immunotherapeutic vaccine for HIV infection. In one of them [178] naked mRNA (iHIVARNA) was administered intranodally to redirect T cell immunity in HIV-infected individuals to the most vulnerable viral targets. iHIVARNA combines an mRNA encoding a novel HIV immunogen sequence (HTI) with TriMix [179], composed by three naked mRNA encoding the DC activation signals CD40L (activation stimuli CD40 ligand), CD70 (costimulatory molecule CD70) and caTLR4 (constitutively active Toll-like receptor 4). iHIVARNA administration was safe, well tolerated and induced moderate HIV-specific T cell responses; however, the phase II study (NCT02888756) was terminated, due to lack of immunogenicity.

Ex vivo strategy for vaccination is based on the use of DCs loaded with mRNA. DCs internalize and process antigens and present them to CD8+ and CD4+ T cells on major histocompatibility complexes (MHCs) class I and II, respectively [164]. DCs are able to internalize naked mRNA by different endocytic ways, although electroporation improves the efficacy. A large number of clinical trials evaluate the use of ex vivo mRNA transfected DCs for cancer, due to the cell-mediated immune response. However, in the case of infectious diseases, this strategy has only been used for HIV immunotherapy (NCT00833781). In this case, intradermal immunization with autologous DCs electroporated with an mRNA encoding HIV-1 Gag and Nef antigens, did not induce significant responses [180].

#### 4.1.2. Cancer Immunotherapy

Cancer immunotherapy relies on the generation of a host anti-tumor immune response, playing a key role cytotoxic T cells, due to their capacity to recognize and kill tumor cells. The induction of a specific immune response by mRNA vaccines begins when the antigen is expressed in the cytosol of antigen-presenting cells or APCs (DCs or macrophages). The resulting proteins are processed by proteasomes and presented on MHC class I molecules to CD8+ T cells, activating cellular response [181]. Complementary, CD4+ T helper cell responses can be triggered by the fusion to the encoded antigen of different trafficking signals of lysosomal proteins residing in MHC class II processing compartments [182,183,184]. Cancer mRNA vaccines present important advantages. On the one band, the possibility of obtaining RNA from a tumor sample to amplify it, yielding large amounts of patient-specific antigens. On the other hand, the capacity of mRNA to act as an adjuvant by providing costimulatory signals, for instance, via TLR3, TLR7 and TLR8 [185].

At present, there is a great number of clinical trials using in vivo or ex vivo mRNA based immunotherapy in various cancer types (Table 2). Although they are in the early stages, encouraging clinical outcomes are expected. The in vivo strategy is based on the direct administration of mRNA, naked or complexed in nanosystems, by different routes, whereas, ex vivo approach is implemented by the administration of IVT mRNA modified DCs or chimeric antigen receptor (CAR) T cells.

Regardless of the strategy, mRNA vaccines can be designed to target a wide variety of antigens, including TAAs, cancer testis antigens (CTAs) and tumor-specific antigens (TSAs); all of them have been tested in clinical trials. TAAs derive from proteins that are overexpressed in cancer cells, but they also occur in normal cells. CTAs are a group of TAAs that might serve as ideal targets for cancer immunotherapy because of their cancer-restricted expression and robust immunogenicity. TSAs derive from viral oncogenic proteins or from proteins produced, due to somatic mutations or gene rearrangements; tumors, in general, acquire mutations during carcinogenesis and progression, resulting in altered proteins that may serve as neoantigens [186]. Potential neoantigen targets, which allow the design of personalized neoepitope cancer vaccines, can be identified after the analysis of the entire somatic cancer mutations in an individual tumor, called mutanome, by means of the whole exome and/or next generation RNA sequencing [187].

Apart from the use of IVT mRNA to produce a specific immune response, non-coding mRNA (CV8102) also has been tested clinically as an adjuvant. CV8102 is a TLR7/8/RIG-1 agonist based on noncoding single stranded RNA, designed to modulate the tumor microenvironment after intratumoral injection. It is being evaluated alone (NCT03291002) or in combination with IMA970A, which is a new cancer vaccine for primary liver cancer based on an off-the-shelf cocktail of 16 peptides (NCT03203005).

The first mRNA cancer vaccine was developed by Conry et al. in 1995 [188]. It consisted of intramuscular injection of mRNA coding for carcinoembryonic antigen (CEA) to mice. This study showed the ability of naked mRNA to induce anti-tumor adaptive immune responses. However, the rapid degradation of mRNA results in low clinical efficiency. Therefore, for in vivo administration, clinical trials usually utilize mRNA formulated in delivery nanosystems. Among them, lipids are the most used followed by polypeptides, and viral vectors have been applied only in one clinical trial. In this sense, in most of the clinical trials that aim to address cancer with personalized vaccines the mRNA encoding antigens are formulated in lipid nanosystems, (NCT03897881, NCT02316457, NCT03313778, NCT03480152, NCT03289962), although naked mRNA also has been evaluated (NCT01684241, NCT02035956).

Besides the delivery system, the administration route also has a significant influence on mRNA vaccine efficacy. Multiple administration approaches have been evaluated in clinical trials to target mRNA to APC cells, from conventional vaccination routes, such as intradermal, intramuscular and subcutaneous, to less common methods, such as intranodal, intravenous or intratumoral. Except for CAR T cells, intradermal route is the most widely used in both, in vivo and ex vivo approaches. Regarding in vivo delivery, CV9201 and CV9202 (NCT00923312 and NCT01915524), based on CureVac’s RNActive^®^ technology, have been applied intradermally for the treatment of non-small cell lung carcinoma (NSCLC). CV9202, containing a sequence-optimized mRNA encoding six NSCLC-associated antigens (NY-ESO-1, MAGE-C1, MAGE-C, Survivin, 5T4, and MUC-1), was well tolerated, and antigen-specific immune responses were detected [189]. Different types of prostate cancers have been addressed by intradermal administration of RNActive^®^ vaccines (NCT01817738, NCT02140138, NCT00831467). In addition, intradermal route has been selected for in vivo strategies against metastatic NSCLC (NCT03164772), malignant melanoma (NCT00204607, NCT03897881), multiple myeloma (NCT01995708), and squamous cell carcinomas (NCT03418480).

Ex vivo therapy with transfected DCs, the most potent APCs of the immune system, is the most frequent strategy used for mRNA cancer vaccination. This method allows precise control of the target cell and transfection efficiency, although it is expensive and laborious. Electroporated DCs with mRNA demonstrated already, in 1996, their capacity to induce an immune response against tumor antigens [190]. Ex vivo strategies generally use intradermal and intravenous routes for DCs and CAR T cells administration, respectively. In 2006, the results of a clinical trial (NCT01278940) that evaluated the intradermal vs. intranodal administration of an individualized melanoma vaccine based on mRNA transfected autologous DCs were published. The vaccine-elicited in vivo T cell responses against expressed tumor antigens, although the response rates did not suggest an advantage in applying intranodal vaccination [191]. TriMixDC-MEL are autologous DCs electroporated with synthetic mRNA TriMix together with mRNA encoding fusion proteins of a human leukocyte antigen (HLA)-class II targeting signal (DC-LAMP), and a melanoma-associated antigen (either MAGE-A3, MAGE-C2, tyrosinase or gp100). Combined intradermal and intravenous administration to patients in stage III or IV melanoma of TriMixDC-MEL resulted in safe, immunogenic and showed anti-tumor activity [192] (NCT01066390). In a later study, patients in stage III or IV melanoma were treated with TriMixDC-MEL combined with the monoclonal antibody Ipilimumab (NCT01302496). This combination was well tolerated and showed a durable tumor reduction in patients with recurrent or refractory melanoma [193]. Apart from melanoma, ex vivo IVT mRNA DCs have been used for other cancers, including acute myeloid leukaemia (AML), multiple myeloma, prostate cancer, glioblastoma and ovarian cancer, among others (Table 2).

Adoptive T cell therapy by ex vivo IVT mRNA based CAR T cell administration has focused great interest thanks to the market of two virally transduced CAR T products in 2017. Tisagenlecleucel and Axicabtagene ciloleucel were approved for the treatment of acute B-cell lymphoblastic leukaemia and large B-cell lymphoma, respectively [194]. Ravinovich et al. [195] transfected for the first time T cells with IVT mRNA encoding CARs against CD19. Transient expression of T cells transduced by IVT mRNA offers safety features and highly efficient recombinant protein translation, but also results in the need for frequent injections of the CAR T cells [196]. Currently, several clinical trials with IVT mRNA CAR T cells for cancer treatment are ongoing. CAR-encoding IVT mRNA targeting the TAA mesothelin was applied against malignant mesothelioma (NCT01355965). However, due to the transient nature of CAR expression on the T cells, repeated infusions were needed, and after the third infusion, one subject developed anaphylaxis and cardiac arrest infusion. These results pointed out a safety issue owing to the potential immunogenicity of CARs derived from murine antibodies, especially when administering intermittently [197]. Application of CD19 CAR T cells targeting the inflammatory tumor microenvironment in Hodgkin’s lymphoma (NCT02277522 and NCT02624258), showed transient responses [198]. Treatment of AML is being attempted by intravenous administration of CD123 CAR T cells. These cells consist of mRNA electroporated autologous T cells expressing anti-CD123 CAR linked to TCR and 4-1BB (TCR/4-1BB) costimulatory signaling domains (NCT02623582). These costimulatory domains are also included in autologous cMet RNA CAR T cells, which has been administered intratumorally in patients with breast cancer, showing good tolerability and evoking an inflammatory response within tumors [199] (NCT01837602). Currently, intravenous administration of cMet RNA CAR T cells in patients with advanced melanoma or breast carcinoma is under evaluation (NCT03060356).

### 4.2. Protein Replacement

The application of IVT mRNA for protein replacement therapies is based on the supplementation of proteins that are infra-expressed or are not functional, as well as on the expression of foreign proteins that can activate or inhibit cellular pathways. Since the first preclinical evaluation of IVT mRNA for protein replacement in 1992 [200], several proteins have been targeted by IVT mRNA administration. In that first study, a temporary reversal of diabetes insipidus was observed after the hypothalamus injection of vasopressin mRNA in Brattleboro rats.

Protein replacement therapies based on IVT mRNA have been mainly directed to the liver [201], lungs [202], and heart [203] because of the accessibility of these organs. However, other organs and tissues, such as skin [204], back of the eye [205] or nasal cavity [131] have also been evaluated as target sites. The main applications of this therapy are genetic and rare diseases. The therapeutic IVT mRNA encodes a missing or down-expressed protein responsible for the disease and associated with genomic defects. These are, for instance, the cases of hemophilia B, characterized by coagulation defects, due to lack of coagulation factor IX [206]; Fabry disease, associated with a deficit of alpha-galactosidase A [207]; methylmalonic acidemia (MMA), caused by methylmalonyl-coenzyme A mutase (MUT) deficiency [208]; propionic acidemia (PA), triggered by a deficiency in the mitochondrial enzyme propionyl-CoA carboxylase [209]; acute intermittent porphyria (AIP), resultant from insufficiency of porphobilinogen deaminase [210]; ornithine transcarbamylase (OTC) deficiency [211]; cystic fibrosis [212] provoked by a genetic defect in the cystic fibrosis transmembrane conductance regulator (CFTR); or William-Beuren syndrome (WBS), which is related to microdeletion of approximately 26 to 28 genes on chromosome 7q11.23, including the elastin gene [204]. Nevertheless, IVT mRNA protein replacement is also useful in disease conditions that are not directly associated with a genetic defect. IVT mRNA encoding vascular endothelial growth factor-A (VEGF-A) may lead to the creation of blood vessels and improve blood supply, providing a regenerative treatment option for patients with ischemic cardiovascular disease, as well as for diabetic wound healing and other ischemic vascular diseases [213,214].

As it is observed in Table 3, several studies have demonstrated the translational potential of IVT mRNA protein replacement therapies to the clinic. In addition, most of them use lipid nanomaterials as delivery systems, which reinforces the importance of an appropriate vehicle to improve efficiency.

Moderna, a clinical-stage biotechnology company pioneering mRNA therapeutics, collaborates with AstraZeneca, a biopharmaceutical company, in two clinical trials in which a naked IVT mRNA encoding VEGF-A (AZD8601) is locally administered in patients undergoing coronary artery bypass grafting surgery with moderately reduced systolic function (NCT03370887) and for the treatment of ulcers in diabetic patients (NCT02935712).

Moderna also has proprietary LNP formulations used as delivery systems in IVT mRNA clinical trials; specifically, for the treatment of PA and MMA, the most common organic acidemias. PA is an autosomal recessive condition caused by mutations in the *PCCA* and *PCCB* genes. These genes encode two subunits of the enzyme propionyl-CoA carboxylase (PPC). Disrupting the function of this enzyme avoids the normal breakdown of proteins and lipids at mitochondrial level. As a result, propionyl-CoA and other toxic compounds accumulate in the body [215]. MMA is also an autosomal recessive disease, which is caused by mutations in the gene coding vitamin B12-dependent enzyme MUT. MUT participates in the intermediary metabolism of proteins, lipids and cholesterol inside the mitochondrial matrix and its deterioration is related to the accumulation of toxic metabolites and to the alteration of mitochondrial oxidative phosphorylation [216]. Currently, there are no approved therapies to treat the underlying cause of PA or MMA; because of the complexity to reach the mitochondrial localization of PPC and MUT, no enzyme replacement therapy is available. The only effective treatment for severely affected individuals is a liver transplant, although it is not a cure because patients remain at risk for disease-related complications [217]. Moderna has recently initiated two clinical trials to evaluate the safety, pharmacokinetics, and pharmacodynamics of different doses of mRNA-3927 (NCT04159103) and mRNA-3704 (NCT03810690) in patients with PA and MMA, respectively. After selecting the dose with satisfactory safety and pharmacodynamic activity, additional patients will be enrolled in a dose expansion phase to allow for further characterization.

Translate Bio, another clinical-stage mRNA therapeutics company, has initiated in the last few years two clinical trials in which synthetic mRNAs, also formulated in LNP, are administered to patients with cystic fibrosis (NCT03375047) or OTC deficiency (NCT03767270). Cystic fibrosis is a monogenic disorder affecting approximately 1 in 2000–3000 newborns in the European Union and 1 in every 3500 births in the United States of America [218]. The disease is caused by genetic variance within the coding region of the CFTR, an anion channel necessary for chloride efflux from secretory epithelial cells. Over time, the resulting ion transport dysregulation induces multisystem organ failure and death [212]. The severity of cystic fibrosis greatly varies from person to person, and it is mainly determined by the degree of the lung affectation. Translate Bio has developed a product known as MRT5005, composed of an IVT mRNA encoding CFTR formulated in LNP developed through its proprietary delivery platform. The results from the single-ascending dose phase of the clinical trial (NCT03375047) in 12 adult patients with cystic fibrosis, who received a single nebulization dose of either MRT5005 or placebo (3:1 randomization), show that the drug was generally well tolerated at the low and mid dose levels. Furthermore, in 4/9 of the subjects treated with MRT5005 the percent predicted forced expiratory volume (ppFEV_1_), a primary measure of lung function, increased [219]. The multiple-ascending dose phase of the clinical trial is ongoing.

OTC deficiency consists of the increment of the ammonia levels in the bloodstream because of disorders in ammonia detoxification and deficiencies of mitochondrial OTC in the urea cycle [220]. Current treatment is based on a protein-restricted diet for life, arginine and citrulline supplementation and currently available ammonia scavengers. However, when there is a critical OTC deficiency in neonatal form or in case of recurrent hyperammonemia episodes, liver transplantation is the only therapeutic option [221]. Translate Bio initiated a clinical trial (NCT03767270) to evaluate the safety, tolerability, and pharmacokinetic/pharmacodynamics of intravenous administration of an IVT mRNA encoding OTC-LNP system (MRT5201). However, it was discontinued because preclinical studies did not support the desired pharmacokinetic and safety profile [222]. The investors relate those preclinical results to the non-optimal features of the first-generation LNPs designed to target the liver; currently, the development of novel next-generation LNPs is supporting the further progress of liver disease IVT mRNA therapeutics.

### 4.3. Gene Editing

Gene editing has recently emerged as a new therapeutic option for a numerous variety of clinical conditions. This technology uses programmable nucleases, engineered to accomplish a DNA double stranded break (DSB) in a specific target location of the genome. The repair of DSBs can be performed by two mechanisms: Homologous-dependent repair (HDR) and non-homologous end joining (NHEJ). In HDR the nucleases act in the presence of a donor DNA template that contains a homologous sequence to be introduced into DSB. This repair is useful to correct genomic mutations or to insert new sequences encoding therapeutic proteins. NHEJ eliminates the target region by binding DSBs; it can be used to silence or correct an anomalous gene [223,224].

There are three main types of gene editing nucleases, composed of a target-sequence-recognizing domain and a nuclease: ZFNs, TALENs, and CRISPR/Cas9.

ZFNs are engineering by fusing zinc-finger DNA binding domains (Cys2-His2) with *Fok*I nuclease as the DNA cleavage domain [225]. In a similar way, TALEN is obtained by a combination of *Fok*I nuclease and the DNA-binding domain derived from transcription activator-like effector (TALE) [226]. In these two strategies, nucleases recognize the target sequences by DNA-protein interaction. The mechanism of action of CRISPR/Cas9 is different; nuclease targeting is mediated through RNA and DNA base pairing. It requires two components: Cas9, a nuclease responsible for the DNA cleavage, and a single guide RNA (sgRNA) that directs Cas9 to cut the DNA at the target sequence [5]. The advantage of CRISPR/Cas9 is that modifying the sequence of sgRNA Cas9 can recognize any sequence of the genome. Additionally, it is possible to edit simultaneously different genes using different sgRNA [227]. However, CRISPR/Cas9 has an important challenge to consider, the complexity of delivering both sgRNA and Cas9.

Gene editing nucleases can be delivered in protein, pDNA or mRNA forms [228]. Delivery in mRNA form offers great potential for therapeutic application. Compared to pDNA, IVT mRNA reduces the risk of genome insertion, and, since the effect is transient, the presence of the nuclease inside the cells and the risk of off-target adverse effects are limited. In contrast to protein delivery, the intracellular presence of the nucleases is more consistent after mRNA expression as compared to the delivery of the nuclease itself [229]. In addition, administration of nucleases in protein form has several limitations for in vivo therapy (cellular and humoral immune response, its charge and large size are challenges for systemic administration, complex protein purification procedures for large nucleases) [228].

IVT mRNA encoding genome-editing nucleases has been mainly used ex vivo to edit T cells or hematopoietic stem and progenitor cells (HSPCs) by electroporation for research and preclinical studies [230,231,232], or to generate animal models by genome editing of embryos or zygotes, by microinjection [227,233,234,235]. Co-delivery of sgRNA and Cas9 mRNA also has been evaluated in vivo in mice, mainly with LNP as delivery systems [73,112,236]. It can be achieved by encapsulation of Cas9 mRNA and sgRNA in two different delivery systems, or by co-formulation into a single nanoparticle [109]. This last strategy ensures simultaneous delivery of these two components to the same individual cell, and it has achieved greater editing efficiency [112].

The translation to clinic of mRNA-based gene editing is currently focused on ex vivo applications (Table 4), especially with ZFN-mRNA and TALEN-mRNA. CRISPR/Cas9-mRNA has been clinically evaluated only in one trial.

Sangamo Therapeutics, a genomic medicine company, is using a ZFN mRNA product (SB-728mR) targeting the human *CCR5* gene in several clinical trials for the treatment of ex vivo HIV-1. C-C chemokine receptor 5 (CCR5) is a co-receptor essential for HIV-1 entry into the cells. The deletion of this protein makes the cells resistant to infection by the virus [237]. Electroporation of SB-728mR is under evaluation in clinical trials to disrupt CCR5 expression in autologous T cells (NCT02225665, NCT04201782), autologous CD4+ T cells (NCT02388594), autologous T cells genetically modified to express a CD4 chimeric antigen receptor (NCT03617198) or autologous CD34+ HSPCs (NCT02500849). In these studies, the cells genetically modified are reinfused intravenously to the patients. Ex vivo ZFN mRNA gene editing is also under clinical evaluation for the treatment of Sickle cell disease. Sickle cell disease or Sickle cell anemia is a globally widespread life-threatening hematological disorder. It is caused by mutations in the hemoglobin genes, resulting in red blood cells with abnormal sickle or crescent shape, which makes them inefficient in their ability to transport oxygen [238]. A clinical study (NCT03653247) is assessing the safety, tolerability, and efficacy of the transplantation in Sickle Cell Disease patients of autologous HSPCs electroporated ex vivo with ZFN mRNAs targeting the B-cell lymphoma/leukemia 11A (*BCL11A*) locus, which plays a role in the fetal to adult erythropoiesis transition [239].

TALEN-mRNA is used to generate UCART19, the first allogenic CAR T cell therapy in clinical study, in pediatric and adult relapsed/refractory B-cell acute lymphoblastic leukemia (NCT02808442, NCT02746952). The base of this product is allogenic T cells engineered to express CARs against the leukemia antigen CD19. In addition, CAR19 T cells are treated by electroporation with TALEN-mRNA to knockout T cell receptors (TCR) and the CD52. The knockout of the TCR is intended to reduce the risk of graft-versus-host disease (GVHD) caused by donor T cells, whereas, knockout of the CD52 gene makes transplanted allogenic T cells resistant to the lymphodepleting agent alemtuzumab [240]. Following a similar strategy, UCART019 is under evaluation in patients with relapsed or refractory CD19+ leukemia and lymphoma (NCT03166878). UCART019 is also based on allogenic CAR19 T cells, but in this case, these cells are treated by CRISPR-Cas9 mRNA electroporation to disrupt endogenous TCR and beta-2 microglobulin (B2M) genes simultaneously, in order to evade host-mediated immunity and to avoid GVHD.

Given the rapid expansion that gene editing is undergoing as a therapeutic tool, and the advantages that mRNA offers in expressing the corresponding nucleases, it is expected that in the near future the number of clinical studies with gene editing mRNA will be significantly increased.

### 4.4. Regenerative Medicine and Cell Engineering

Regenerative medicine aims to regrow, repair or replace injured or lost cells, organs or tissues by restoring or establishing their normal function [241]. The regeneration process needs the functionality of proteins, such as growth factors, cytokines and transcription factors, which control cellular biological activity, including cell mitosis, migration or differentiation. Among the regenerative strategies, reprogramming and transdifferentiation of somatic cells into other specific lineages can help to repair cell or tissue deficits in patients. In both processes specific transcription factors are required to bind to enhancer or promoter sequences of DNA and regulate gene expression. However, the intracellular delivery of the transcription factors is quite limited by the plasmatic membrane [15,51], and the use of pDNA to express them also shows the risk of the insertion and of the consequent mutagenesis. Transfection of somatic cells with IVT mRNA encoding transcription factors has been assessed as an attractive alternative to conventional reprogramming and transdifferentiation of somatic cells [51], although research in this field is quite incipient and, up to date, it is limited to in vitro preclinical works.

**Figure 5:**
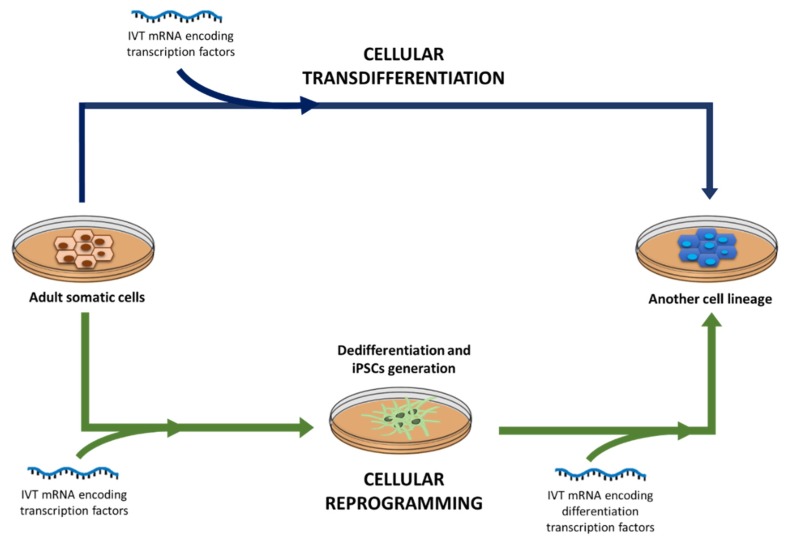
Representative scheme of cellular transdifferentiation and cellular reprogramming therapies.

Cellular reprogramming is based on the generation of iPSCs from a patient’s adult somatic cells. Those iPSCs will be later differentiated into autologous specific cell types [5,15,51]. Alternatively, in the transdifferentiation or direct reprogramming process, adult somatic cells are transformed into cell lineages without iPSC generation [242,243]. Figure 5 depicts the differences between cellular reprogramming and transdifferentiation. Depending on the cocktail of transcription factors expressed by the IVT mRNA and on the adult somatic cell type, reprogramming or transdifferentiation will be performed, and different lineages can be obtained [155,244,245,246,247].

In 2010, it was reported the first study of in vitro reprogramming of somatic cells by IVT mRNA to mediate was reported. iPSCs were generated by delivering synthetic mRNA, encoding four transcription factors (Oct4, Sox2, Lin28, and Nanog) to human foreskin fibroblasts [244]. In the same year, another study reported the use of IVT mRNA encoding the following mix of transcription factors, Oct4, Sox2, Klf4, and cMyc (known as Yamanaka factors [245]), to obtain iPSCs from fibroblasts [246]. In this study, additionally, the iPSCs were differentiated toward myofibers after transfection with myogenic differentiation factor (MyoD) IVT mRNA.

Transdifferentiation of somatic cells with IVT mRNA has been mainly focused on the generation of insulin secreting β-cells for type 1 diabetes patients [247,248,249], and on obtaining cardiomyocytes to regenerate the cardiac tissue after a heart attack [155]. mRNA is a promising tool for the delivery of factors targeting altered signaling pathways in the early hours of infarction, as well as to address experimental and clinical needs to regenerate cardiac tissue and cardiac function in ischemic heart disease.

mRNA therapeutics also show significant potential in stimulating bone regeneration. Transcript Activated Matrices (TAMs), have been evaluated as platforms for sustained delivery of mRNA encoding osteogenic proteins (i.e., BMP-2), while supporting cell proliferation, extracellular matrix deposition and ultimately de novo tissue formation. TAMs provide steady state protein production for up to 6 days, and substantial residual expression until 11 days after transfection [250,251]

Another application of IVT mRNA in regenerative medicine is mesenchymal stem cells (MSCs) engineering. MSCs are adult stem cells that can be isolated from different sources, including bone marrow, umbilical cord, adipose tissue, liver, multiple dental tissues, and iPSCs [252]. Among their properties, MSCs show migration capacity and the ability of self-renewal and differentiation into a wide range of cell lines, such as cartilage, adipocytes, and bone. In addition, MSCs secrete a huge variety of proteins which can promote the angiogenesis, reduce inflammation and improve tissue reparation. Engineering of MSCs with IVT mRNA has been proposed to modulate the migratory properties of these cells, by expressing homing proteins in a brief, burst and temporary way. It is advantageous because MSCs treated with other strategies of cell surface modification (enzymatic or chemical modification and DNA-based genetic modifications) may suffer permanent changes by alterations in the cell membrane, which, in turn, result in disorders of the differentiation properties [51].

IVT mRNA-based modulation of homing MSCs to target ischemic areas has been assessed by in vitro transfection of different surface molecules, including the chemokine receptor CXCR4 [253], and the integrin α4 (ITGA4) [254]. MSCs may be also used as a vehicle to target biological molecules, such as interleukins (IL), to specific tissues. In this sense, Levy et al. [255] transfected MSCs by lipofection with the IVT mRNA encoding the adhesion molecules P-selectin glycoprotein ligand-1 (PSGL-1) and Sialyl-Lewis^x^ (SLeX), as well as, the anti-inflammatory molecule IL-10. After intravenous administration of the engineered MSCs to C57BL/6 mice with induced ear inflammation, the authors demonstrated the migration capacity of the cells and a high level of IL-10 expression in the target tissue.

Increasingly the number of mRNA studies are attempting to validate proof-of-concept in regenerative medicine; the encouraging results suggest that mRNA-based therapies will direct the incoming direction of tissue regeneration.

## 5. Conclusions

Synthetic mRNA is attracting great interest as a therapeutic molecule. The main feature that has encouraged its recent expansion is the controlled expression of transgenes without the risk of insertional mutagenesis or permanent genomic alteration. Other advantages include economic production, scalable manufacturing, and versatility of applications. Limiting technological issues mainly associated with delivery and stability difficulties still have to be overcome, although important advances have been made in the last years.

Innovative progress in IVT mRNA design, as well as significant advances in nanodelivery systems, are approaching its clinical translation. Unlike DNA gene therapy, non-viral vectors are at the forefront of IVT mRNA therapy, and among them, LNP have the best prospects for mRNA-based medicines development. The combination of IVT mRNA and nanotechnology, as two powerful technological tools, is undergoing significant growth, and it is expected to play a key role in the biotechnology industry in the near future.

Gene editing, protein replacement therapy and immunotherapy have entered the early phases of clinical trials, whereas, cellular reprogramming and engineering are still in preclinical stages. Immunotherapy for prophylaxis and therapy of infectious diseases has shown the high influence of the delivery system and the administration route on the efficacy. Most clinical trials are focused on cancer immunotherapy; actually, the therapeutic perspectives of this field have been broadening, thanks to the beginning of the first mRNA CAR T cells clinical trials. Encouraging outcomes are expected, although the results of ongoing and future clinical research will help to define more accurately the therapeutic potential of mRNA.

## Figures and Tables

**Table 1:** Clinical Trials of mRNA vaccines against infectious diseases.

Infectious Disease	Biological Active/Encoding Sequence	Strategy/Delivery System	Administration Route	NCT Number/Phase
Rabies	CV7201 mRNA/Rabies virus glycoprotein (RABV-G)	In vivo/Polypeptide system	i.d. or i.m.	NCT02241135/Phase I
	CV7202 mRNA/Rabies virus glycoprotein (RABV-G)	In vivo/Lipid nanosystem	i.m.	NCT03713086/Phase I
Zika Virus	mRNA-1893/Structural proteins of Zika virus	In vivo/Lipid nanosystem		NCT04064905/Phase I
	mRNA-1325/Zika virus antigen	In vivo/Lipid nanosystem	i.d.	NCT03014089/Phase I
Cytomegalovirus (CMV)	mRNA-1647 and mRNA-1443/Pentamer complex and full-length membrane-bound glycoprotein B (gB) and pp65 T cell antigen of CMV	In vivo/Lipid nanosystem	i.d.	NCT03382405/Phase I
	AVX601/Alphavirus replicon vaccine expressing gB, pp65 and IE1 proteins of CMV	In vivo/Viral vector	i.m. or s.c.	NCT00439803/Phase I
hMPV and PIV3	mRNA-1653: Fusion proteins of hMPV and PIV3	In vivo/Lipid nanosystems	i.d.	NCT03392389/Phase I
Tuberculosis	GSK 692,342/Immunogenic fusion protein (M72) derived from Mycobacterium tuberculosis	In vivo/Lipid nanosytems	i.m.	NCT01669096/Phase II
HIV-1	HIV-1 Gag and Nef	Ex vivo/mRNA transfected autologous DCs	i.d.	NCT00833781/Phase I
	iHIVARNA: TriMix and HTI/APC activation molecules (CD40L+CD70+caTLR4) and HIV immunogen sequences (Gag, Pol, Vif and Nef)	In vivo/Naked mRNA	Inguinal intranodal	NCT02413645 and NCT02888756/Phase I and Phase II
	AVX101/Alphavirus replicon vaccine expressing HIV Gag antigen	In vivo/Viral vector	s.c.	NCT00097838 and NCT00063778/Phase I
Influenza	VAL-506440/H10N8 antigen	In vivo/Lipid nanosystems	i.d. or i.m.	NCT03076385/Phase I
	VAL-339851/H7N9 antigen	In vivo/Lipid nanosystems	i.d. or i.m.	NCT03345043/Phase I
	AVX502/Alphavirus replicon vaccine expressing Influenza A/Wyoming/03/2003 Hemagglutinin	In vivo/Viral vector	i.m. or s.c.	NCT00440362 and NCT00706732/Phase I/II

DCs, dendritic cells; i.d., intradermal; i.m., intramuscular; s.c., subcutaneous; i.v., intravenous; hMPV, human metapneumovirus; PIV3, human parainfluenza virus type 3; HIV-1, human immunodeficiency virus type 1; APC, antigen-presenting cells.

**Table 2:** Clinical trials of mRNA for cancer immunotherapy.

Type of Cancer	Biological Active/Encoding Sequence	Strategy/Delivery System	Administration Route	NCT Number/Phase
Non-small-cell lung carcinoma (NSCLC)	CV9201/five mRNAs encoding antigens which are overexpressed or exclusively expressed in NSCLC cells	In vivo/Polypeptide system	i.d.	NCT00923312/Phase I/II
	CV9202/six mRNAs encoding antigens which are overexpressed in NSCLC compared to healthy tissue	In vivo/Polypeptide system	i.d.	NCT01915524/Phase I
Metastatic NSCLC	BI 1,361,849/NSCLC-associated antigens (NY-ESO-1, MAGE-C1, MAGE-C2, 5T4, and MUC-1)	In vivo/Polypeptide system	i.d.	NCT03164772/Phase I and II
Esophageal Cancer and NSCLC	Personalized mRNA Tumor Vaccine/Neoantigen (tumor associated specific antigens)	In vivo/-	s.c.	NCT03908671/NotA
Malignant Melanoma	mRNA coding for melanoma associated antigens	In vivo/Naked mRNA	s.c.	NCT00204516/Phase I/II
	mRNA coding melanoma associated antigens (Melan-A, Mage-A1, Mage-A3, Survivin, GP100 and Tyrosinase)	In vivo/Polypeptide system	i.d.	NCT00204607/Phase I/II
	mRNA coding the unique spectrum of tumor antigens in each patient	Ex vivo/mRNA transfected DCs	i.d. or intranodal	NCT01278940/Phase I/II
Malignant Melanoma III and IV	TriMix-DC encoding melamona tumor-associated antigens (MAGE-A3, MAGE-C2, tyrosinase and gp100)	Ex vivo/autologous TriMix-DC	i.v.	NCT01302496/Phase II
Melanoma	mRNA-4157/personalized cancer vaccine targeting twenty tumor-associated antigens	In vivo/Lipid nanosystems	i.d.	NCT03897881/Phase II
	(RBL001; RBL002)/malignant melanoma associated antigens	In vivo/Naked mRNA	intranodal	NCT01684241/Phase I
	IVAC MUTANOME/poly-neo-epitopic personalized cancer vaccine targeting tumor-associated antigens (with or without initial treatment with RBL001/RBL002)	In vivo/Naked mRNA	intranodal	NCT02035956/Phase I
	RBL001.1; RBL002.2; RBL003.1; RBL004.1/malignant melanoma-associated antigens	In vivo/Lipid nanosystems	i.v.	NCT02410733/Phase I
	mRNA encoding TriMix	Ex vivo/mRNA-transfected autologous DCs	i.d. and i.v.	NCT01066390/Phase I
	mRNA encoding melanoma-associated tumor antigens (gp100 and tyrosinase) and TriMix	Ex vivo/mRNA-transfected autologous DCs	intranodal	NCT01530698/Phase I/II
Melanoma Stage III or IV	mRNA encoding melanoma associated antigens (gp100 and tyrosinase)	Ex vivo/mRNA-transfected DCs	i.v., i.d., intranodal	NCT00243529/Phase I/II
Metastatic Malignant Melanoma	hTERT-, Survivin- and tumor cell derived mRNA + ex vivo T cell expansion	Ex vivo/mRNA-transfected DCs	i.d. and i.v.	NCT00961844/Phase I/II
Uveal Melanoma	mRNA coding tumor associated antigens	Ex vivo/mRNA-transfected DCs	i.d./i.v.	NCT00929019/Phase I/II
Acute Myeloid Leukemia (AML)	mRNA coding for the Wilms’ tumor protein (WT1)	Ex vivo/mRNA transfected autologous DCs	i.d.	NCT00834002/Phase I
	AML-specific mRNA	Ex vivo/mRNA transfected autologous DCs	i.d.	NCT00514189/Phase I
	mRNA encoding WT1, PRAME, and CMVpp65	Ex vivo/mRNA transfected autologous DCs	i.d.	NCT01734304/Phase I/II
Relapsed or Refractory AML	Autologous Anti-CD 123 CAR TCR/4-1BB-expressing T-lymphocytes/anti-CD123 chimeric antigen receptors expressing tandem TCR and 4-1BB (TCR/4-1BB) costimulatory domains	Ex vivo/mRNA transfected autologous CAR T cells	iv	NCT02623582/Early Phase I
Multiple Myeloma	mRNA encoding CT7, MAGE-A3, and WT1	Ex vivo/mRNA-transfected autologous Langerhans-type DCs	i.d.	NCT01995708/Phase I
Prostate Cancer	CV9104/mRNAs encoding PSA, PSCA, PSMA, STEAP1, PAP and Mucin 1 antigens	In vivo/Polypeptide system	i.d.	NCT01817738 and NCT02140138/Phase I/II and Phase II
	mRNA coding tumor associated antigens	Ex vivo/mRNA-transfected DCs	i.d.	NCT01278914/Phase I/II
	mRNA extracted from Primary Prostate Cancer Tissue, combined with mRNA encoding hTERT and Survivin	Ex vivo/mRNA-transfected DCs	i.d.	NCT01197625/Phase I/II
Metastatic Prostate Cancer	mRNA derived from the patient’s own tumor	Ex vivo/mRNA-transfected autologous DCs	i.d.	NCT01153113/Phase I/II (withdrawn)
Hormonal Refractory Prostate Cancer	CV9103/mRNAs encoding PSA, PSCA, PSMA and STEAP1 antigens	In vivo/Polypeptide system	i.d.	NCT00831467 (eudract 2008-003967-37) and NCT00906243/Phase I/II
Glioblastoma	mRNA encoding Survivin, hTERT or autologous tumor stem cells derived from tumorspheres	Ex vivo/mRNA-transfected autologous DCs	id	NCT03548571/Phase II/III
Ovarian Cancer	W_ova1 vaccine: Three ovarian cancer tumor associated antigens mRNAs	In vivo/Lipid nanosystems	i.v.	NCT04163094/Phase I
Recurrent Epithelial Ovarian Cancer	mRNA encoding hTERT and Survivin in addition to amplified cancer stem cell mRNA	Ex vivo/mRNA-transfected DCs	i.d.	NCT01334047/Phase I/II
Breast Cancer	cMet RNA CAR T cells	Ex vivo/mRNA transfected autologous CAR T cells	intratumoral	NCT01837602/Phase I
Early Breast Cancer	mRNA encoding TriMix	In vivo/naked mRNA	intratumoral	NCT03788083/Phase I
Triple-negative breast cancer	IVAC_WAREHOUSE_bre1_uID; IVAC MUTANOME _uID/personalized cancer vaccine targeting tumor-associated antigens	In vivo/Lipid nanosystems	i.v.	NCT02316457/Phase I
Solid tumors	mRNA-4157/personalized cancer vaccine targeting twenty tumor-associated antigens	In vivo/Lipid nanosystem	i.m.	NCT03313778/Phase I
Hodgkin Lymphoma	RNA anti-CD19 CAR T cells/CD19 chimeric antigen receptors expressing tandem TCR/4-1BB costimulatory domains	Ex vivo/mRNA transfected autologous CAR T cells	i.v.	NCT02277522 and NCT02624258/Early Phase I
Metastatic Pancreatic Ductal Adenocarcinoma	RNA mesothelin re-directed autologous T cell/chimeric anti-mesothelin immunoreceptor SS1	Ex vivo/mRNA transfected autologous CAR T cells	i.v.	NCT01897415/Phase I
Malignant Pleural Mesothelioma	Autologous anti-mesothelin CAR T cells/chimeric anti-mesothelin immunoreceptor	Ex vivo/mRNA transfected autologous CAR T cells	i.v.	NCT01355965/Phase I
Malignant Melanoma, Breast Cancer	RNA CART-cMET/MET chimeric antigen receptors with tandem TCRζ and 4-1BB (TCRζ/4-1BB) co-stimulatory domains	Ex vivo/mRNA transfected autologous CAR T cells	i.v.	NCT03060356/Early Phase I
Brain Cancer, Neoplasm Metastases	Personalized cellular vaccine/tumor associated antigen mRNA	Ex vivo/mRNA transfected autologous DCs	NA	NCT02808416/Phase I
Advanced Esophageal Squamous Carcinoma, Gastric Adenocarcinoma, Pancreatic Adenocarcinoma, Colorectal Adenocarcinoma	Personalized mRNA Tumor Vaccine/Neoantigen (tumor associated specific antigens)	In vivo/-	s.c.	NCT03468244/NotA
Melanoma, Colon cancer, Gastrointestinal cancer, Genitourinary cancer, hepatocellular cancer	NCI-4650/mRNA-based, Personalized Cancer Vaccine	In vivo/Lipid nanosystems	i.m.	NCT03480152/Phase I/II
Melanoma, NSCLC, Bladder Cancer, Colorectal Cancer, Triple Negative Breast Cancer, Renal Cancer, Head and Neck Cancer, Other Solid Cancers	RO7198457/personalized cancer vaccine targeting tumor-associated antigens	In vivo/Lipid nanosystem	i.v.	NCT03289962/Phase I
Relapsed/Refractory Solid Tumor Malignancies or Lymphoma, Ovarian Cancer	mRNA-2416/OX40L	In vivo/Lipid nanosystems	Intratumoral	NCT03323398/Phase I and II
Squamous Cell Carcinoma, Head and Neck Neoplasm, Cervical Neoplasm, Penile Neoplasms Malignant	human papillomavirus (HPV16) mRNA vaccine/HPV16-derived E6, E7 tumor antigens	In vivo/Naked mRNA	i.d.	NCT03418480/Phase I and II
Advanced or Metastatic Malignancies Expressing CEA (Colorectal Cancer, Breast Cancer, Lung Cancer, Pancreatic Cancer) or Stage III Colon Cancer	AVX701/Alphaviral replicon particle vaccine expressing Carcinoembryonic Antigen Gene (CEA(6D)).	In vivo/Viral vector	i.m.	NCT00529984, NCT01890213/Phase I and II, Phase I
Glioblastoma, Renal Cell Carcinoma, Sarcomas, Breast Cancers, Malignant Mesothelioma, Colorectal Tumor	mRNA encoding WT1	Ex vivo/mRNA-transfected autologous DCs	i.d.	NCT01291420/Phase I/I,

NA, not available; NotA, not applicable; DCs, dendritic cells; i.d., intradermal; s.c., subcutaneous; i.v., intravenous; i.m., intramuscular.

**Table 3:** Clinical trials of mRNA for protein-replacement therapies.

Disease	Biological Active/Encoding Sequence	Strategy/Delivery System	Administration Route	NCT Number/Phase
Heart Failure	AZD8601/Vascular endothelial growth factor-A (VEGF-A)	Naked mRNA	Epicardial injection	NCT03370887/Phase II
Ulcers associated with type II diabetes	AZD8601/Vascular endothelial growth factor-A (VEGF-A)	Naked mRNA	Intradermal	NCT02935712/Phase I
Propionic Acidemia	mRNA-3927/alpha and beta subunits of the mitochondrial enzyme propionyl-CoA carboxylase	In vivo/Lipid nanosytems	Intravenous	NCT04159103/Phase I and II
Isolated Methylmalonic Acidemia	mRNA-3704/methylmalonyl-coenzyme A mutase (MUT)	In vivo/Lipid nanosytems	Intravenous	NCT03810690/Phase I and II
Ornithine Transcarbamylase Deficiency	MRT5201/Ornithine transcarbamylase	In vivo/Lipid nanosystems	Intravenous	NCT03767270/Phase I and II
Cystic Fibrosis	MRT5005/Human Cystic Fibrosis Transmembrane Regulator protein (CFTR)	In vivo/Lipid nanosystems	Nebulization	NCT03375047/Phase I and II

**Table 4:** Clinical trials of mRNA for gene editing therapy.

Disease	Biological Active	Therapeutic mRNA	Target Protein	Strategy/Delivery System	Administration Route	NCT Number/Phase
HIV	SB-728mR	ZFN mRNA	CCR5	Ex vivo/Autologous CD4+ T Cells	Intravenous	NCT02388594/Phase I
	SB-728mR	ZFN mRNA	CCR5	Ex vivo/Autologous CD4 CAR+ T cells	Intravenous	NCT03617198/Phase I
	SB-728mR-T	ZFN mRNA	CCR5	Ex vivo/Autologous T cells	Intravenous	NCT02225665, NCT04201782,/Phase I, Phase I/II
	SB-728mR-HSPC	ZFN mRNA	CCR5	Ex vivo/Autologous CD34+ hHSPCs	Intravenous	NCT02500849/Phase I
Sickle Cell Disease	BIVV003	ZFN mRNA	B-cell lymphoma/leukemia 11A (BCL11A)	Ex vivo/Autologous CD34 + hematopoietic stem cells (HSPC)	Intravenous	NCT03653247/Phase I/II
B acute lymphoblastic leukemia	UCART19	TALEN mRNA	TCR and CD52	Ex vivo/Allogenic T cells	Intravenous	NCT02808442, NCT02746952, NCT02735083/Phase I
B cell leukemia and B cell lymphoma	UCART019	CRISPR/Cas9 mRNA	TCR, B2M	Ex vivo/Allogenic T cells	Intravenous	NCT03166878/Phase I/II

## References

[1] European Medicines Agency (2015). Guideline on the quality, non-clinical and clinical aspects of gene therapy medicinal products. Eur. Med. Agency Guidel..

[2] del Pozo-Rodríguez A., Rodríguez-Gascón A., Rodríguez-Castejón J., Vicente-Pascual M., Gómez-Aguado I., Battaglia L.S., Solinís M.A. (2019). Gene therapy. Adv. Biochem. Eng. Biotechnol..

[3] Thorne B., Takeya R., Vitelli F., Swanson X., Kiss B., Gottschalk U., Pohlscheidt M. (2017). Gene Therapy. New Bioprocessing Strategies: Development and Manufacturing of Recombinant Antibodies and Proteins. Advances in Biochemical Engineering/Biotechnology.

[4] Anguela X.M., High K.A. (2019). Entering the Modern Era of Gene Therapy. Ann. Rev. Med..

[5] Hajj K.A., Whitehead K.A. (2017). Tools for translation: Non-viral materials for therapeutic mRNA delivery. Nat. Rev. Mater..

[6] Sahin U., Karikó K., Türeci Ö. (2014). MRNA-based therapeutics-developing a new class of drugs. Nat. Rev. Drug Discov..

[7] Meng Z., O’Keeffe-Ahern J., Lyu J., Pierucci L., Zhou D., Wang W. (2017). A new developing class of gene delivery: Messenger RNA-based therapeutics. Biomater. Sci..

[8] Zarghampoor F., Azarpira N., Khatami S.R., Behzad-Behbahani A., Foroughmand A.M. (2019). Improved translation efficiency of therapeutic mRNA. Gene.

[9] Zhong Z., Mc Cafferty S., Combes F., Huysmans H., De Temmerman J., Gitsels A., Vanrompay D., Portela Catania J., Sanders N.N. (2018). mRNA therapeutics deliver a hopeful message. Nano Today.

[10] Schlake T., Thess A., Thran M., Jordan I. (2019). mRNA as novel technology for passive immunotherapy. Cell. Mol. Life Sci..

[11] Patel S., Athirasala A., Menezes P.P., Ashwanikumar N., Zou T., Sahay G., Bertassoni L.E. (2019). Messenger RNA Delivery for Tissue Engineering and Regenerative Medicine Applications. Tissue Eng. Part A.

[12] Hecker J.G., Manfredsson F.P. (2016). Non-Viral, Lipid-Mediated DNA and mRNA Gene Therapy of the Central Nervous System (CNS): Chemical-Based Transfection. Gene Therapy for Neurological Disorders: Methods and Protocols.

[13] Vallazza B., Petri S., Poleganov M.A., Eberle F., Kuhn A.N., Sahin U. (2015). Recombinant messenger RNA technology and its application in cancer immunotherapy, transcript replacement therapies, pluripotent stem cell induction, and beyond. Wiley Interdiscip. Rev. RNA.

[14] Kowalski P.S., Rudra A., Miao L., Anderson D.G. (2019). Delivering the Messenger: Advances in Technologies for Therapeutic mRNA Delivery. Mol. Ther..

[15] Xiong Q., Lee G.Y., Ding J., Li W., Shi J. (2018). Biomedical applications of mRNA nanomedicine. Nano Res..

[16] del Pozo-Rodríguez A., Delgado D., Solinís M.A., Gascón A.R., Pedraz J.L. (2008). Solid lipid nanoparticles for retinal gene therapy: Transfection and intracellular trafficking in RPE cells. Int. J. Pharm..

[17] Gan L., Wang J., Zhao Y., Chen D., Zhu C., Liu J., Gan Y. (2013). Hyaluronan-modified core-shell liponanoparticles targeting CD44-positive retinal pigment epithelium cells via intravitreal injection. Biomaterials.

[18] Apaolaza P.S., del Pozo-Rodríguez A., Solinís M.A., Rodríguez J.M., Friedrich U., Torrecilla J., Weber B.H.F., Rodríguez-Gascón A. (2016). Structural recovery of the retina in a retinoschisin-deficient mouse after gene replacement therapy by solid lipid nanoparticles. Biomaterials.

[19] Stewart M.P., Sharei A., Ding X., Sahay G., Langer R., Jensen K.F. (2016). In vitro and ex vivo strategies for intracellular delivery. Nature.

[20] Patel S., Ashwanikumar N., Robinson E., Duross A., Sun C., Murphy-Benenato K.E., Benenato M., Mihai C., Almarsson O., Sahay G. (2017). Boosting Intracellular Delivery of Lipid Nanoparticle-Encapsulated mRNA. Nano Lett..

[21] Steinle H., Behring A., Schlensak C., Wendel H.P., Avci-Adali M. (2017). Concise Review: Application of In Vitro Transcribed Messenger RNA for Cellular Engineering and Reprogramming: Progress and Challenges. Stem Cells.

[22] Karikó K., Muramatsu H., Ludwig J., Weissman D. (2011). Generating the optimal mRNA for therapy: HPLC purification eliminates immune activation and improves translation of nucleoside-modified, protein-encoding mRNA. Nucleic Acids Res..

[23] Weissman D. (2014). mRNA transcript therapy. Expert Rev. Vaccines.

[24] Islam M.A., Reesor E.K.G., Xu Y., Zope H.R., Zetter B.R., Shi J. (2015). Biomaterials for mRNA delivery. Biomater. Sci..

[25] Ramanathan A., Robb G.B., Chan S.H. (2016). mRNA capping: Biological functions and applications. Nucleic Acids Res..

[26] Muttach F., Muthmann N., Rentmeister A. (2017). Synthetic mRNA capping. Beilstein J. Org. Chem..

[27] Werner M., Purta E., Kaminska K.H., Cymerman I.A., Campbell D.A., Mittra B., Zamudio J.R., Sturm N.R., Jaworski J., Bujnicki J.M. (2011). 2′-O-ribose methylation of cap2 in human: Function and evolution in a horizontally mobile family. Nucleic Acids Res..

[28] McCracken S., Fong N., Rosonina E., Yankulov K., Brothers G., Siderovski D., Hessel A., Foster S., Shuman S., Bentley D.L. (1997). 5′-Capping enzymes are targeted to pre-mRNA by binding to the phosphorylated carboxy-terminal domain of RNA polymerase II. Genes Dev..

[29] Fuchs A.-L., Neu A., Sprangers R. (2016). A general method for rapid and cost-efficient large-scale production of 5′ capped RNA. RNA.

[30] Trilink Biotechnologies. https://www.trilinkbiotech.com/cleancap.

[31] Warminski M., Kowalska J., Buck J., Zuberek J., Lukaszewicz M., Nicola C., Kuhn A.N., Sahin U., Darzynkiewicz E., Jemielity J. (2013). The synthesis of isopropylidene mRNA cap analogs modified with phosphorothioate moiety and their evaluation as promoters of mRNA translation. Bioorg. Med. Chem. Lett..

[32] Ziemniak M., Kowalska J., Lukaszewicz M., Zuberek J., Wnek K., Darzynkiewicz E., Jemielity J. (2015). Phosphate-modified analogues of m7GTP and m7Gppppm7G - Synthesis and biochemical properties. Bioorg. Med. Chem..

[33] Kuhn A.N., Diken M., Kreiter S., Selmi A., Kowalska J., Jemielity J., Darzynkiewicz E., Huber C., Türeci Ö., Sahin U. (2010). Phosphorothioate cap analogs increase stability and translational efficiency of RNA vaccines in immature dendritic cells and induce superior immune responses in vivo. Gene Ther..

[34] Strenkowska M., Kowalska J., Lukaszewicz M., Zuberek J., Su W., Rhoads R.E., Darzynkiewicz E., Jemielity J. (2010). Towards mRNA with superior translational activity: Synthesis and properties of ARCA tetraphosphates with single phosphorothioate modifications. New J. Chem..

[35] Zytek M., Kowalska J., Lukaszewicz M., Wojtczak B.A., Zuberek J., Ferenc-Mrozek A., Darzynkiewicz E., Niedzwiecka A., Jemielity J. (2014). Towards novel efficient and stable nuclear import signals: Synthesis and properties of trimethylguanosine cap analogs modified within the 5′,5′-triphosphate bridge. Org. Biomol. Chem..

[36] Rydzik A.M., Kulis M., Lukaszewicz M., Kowalska J., Zuberek J., Darzynkiewicz Z.M., Darzynkiewicz E., Jemielity J. (2012). Synthesis and properties of mRNA cap analogs containing imidodiphosphate moiety—Fairly mimicking natural cap structure, yet resistant to enzymatic hydrolysis. Bioorg. Med. Chem..

[37] Al-Saif M., Khabar K.S.A. (2012). UU/UA dinucleotide frequency reduction in coding regions results in increased mRNA stability and protein expression. Mol. Ther..

[38] Mauro V.P., Chappell S.A. (2014). A critical analysis of codon optimization in human therapeutics. Trends Mol. Med..

[39] Alexaki A., Hettiarachchi G.K., Athey J.C., Katneni U.K., Simhadri V., Hamasaki-Katagiri N., Nanavaty P., Lin B., Takeda K., Freedberg D. (2019). Effects of codon optimization on coagulation factor IX translation and structure: Implications for protein and gene therapies. Sci. Rep..

[40] Presnyak V., Alhusaini N., Chen Y.H., Martin S., Morris N., Kline N., Olson S., Weinberg D., Baker K.E., Graveley B.R. (2015). Codon optimality is a major determinant of mRNA stability. Cell.

[41] Hunt R.C., Simhadri V.L., Iandoli M., Sauna Z.E., Kimchi-Sarfaty C. (2014). Exposing synonymous mutations. Trends Genet..

[42] McCarthy C., Carrea A., Diambra L. (2017). Bicodon bias can determine the role of synonymous SNPs in human diseases. BMC Genom..

[43] Bali V., Bebok Z. (2015). Decoding mechanisms by which silent codon changes influence protein biogenesis and function. Int. J. Biochem. Cell Biol..

[44] Li B., Zhang X., Dong Y. (2019). Nanoscale platforms for messenger RNA delivery. Wiley Interdiscip. Rev. Nanomed. Nanobiotechnol..

[45] Jalkanen A.L., Coleman S.J., Wilusz J. (2014). Determinants and implications of mRNA poly(A) tail size—Does this protein make my tail look big. Seminars in Cell & Developmental Biology.

[46] Eckmann C.R., Rammelt C., Wahle E. (2011). Control of poly(A) tail length. Wiley Interdiscip. Rev. RNA.

[47] Weill L., Belloc E., Bava F.A., Méndez R. (2012). Translational control by changes in poly(A) tail length: Recycling mRNAs. Nat. Struct. Mol. Biol..

[48] Peng J., Schoenberg D.R. (2005). mRNA with a <20-nt poly(A) tail imparted by the poly(A)-limiting element is translated as efficiently in vivo as long poly(A) mRNA. RNA.

[49] Holtkamp S., Kreiter S., Selmi A., Simon P., Koslowski M., Huber C., Türeci O., Sahin U. (2006). Modification of antigen-encoding RNA increases stability, translational efficacy, and T-cell stimulatory capacity of dendritic cells. Blood.

[50] Mockey M., Gonçalves C., Dupuy F.P., Lemoine F.M., Pichon C., Midoux P. (2006). mRNA transfection of dendritic cells: Synergistic effect of ARCA mRNA capping with Poly(A) chains in cis and in trans for a high protein expression level. Biochem. Biophys. Res. Commun..

[51] Kwon H., Kim M., Seo Y., Moon Y.S., Lee H.J., Lee K., Lee H. (2018). Emergence of synthetic mRNA: In vitro synthesis of mRNA and its applications in regenerative medicine. Biomaterials.

[52] Matoulkova E., Michalova E., Vojtesek B., Hrstka R. (2012). The role of the 3′ untranslated region in post-transcriptional regulation of protein expression in mammalian cells. RNA Biol..

[53] Hellen C.U.T., Sarnow P. (2001). Internal ribosome entry sites in eukaryotic mRNA molecules. Genes Dev..

[54] Johnson A.G., Grosely R., Petrov A.N., Puglisi J.D., Puglisi J.D. (2017). Dynamics of IRES-mediated translation. Philos. Trans. R. Soc. B Biol. Sci..

[55] Yang Y., Wang Z. (2019). IRES-mediated cap-independent translation, a path leading to hidden proteome. J. Mol. Cell Biol..

[56] Kozak M. (1984). Point mutations close to the AUG initiator codon affect the efficiency of translation of rat preproinsulin in vivo. Nature.

[57] Kozak M. (1987). At least six nucleotides preceding the AUG initiator codon enhance translation in mammalian cells. J. Mol. Biol..

[58] Jiang Y., Xu X.-S., Russell J.E. (2006). A Nucleolin-Binding 3′ Untranslated Region Element Stabilizes β-Globin mRNA In Vivo. Mol. Cell. Biol..

[59] Volloch V., Housman D. (1981). Stability of globin mRNA in terminally differentiating murine erythroleukemia cells. Cell.

[60] Berkovits B.D., Mayr C. (2015). Alternative 3′ UTRs act as scaffolds to regulate membrane protein localization. Nature.

[61] Karikó K., Muramatsu H., Welsh F.A., Ludwig J., Kato H., Akira S., Weissman D. (2008). Incorporation of pseudouridine into mRNA yields superior nonimmunogenic vector with increased translational capacity and biological stability. Mol. Ther..

[62] Hornung V., Ellegast J., Kim S., Brzózka K., Jung A., Kato H., Poeck H., Akira S., Conzelmann K.-K., Schlee M. (2006). 5′-Triphosphate RNA Is the Ligand for RIG-I. Science.

[63] Diebold S.S., Massacrier C., Akira S., Paturel C., Morel Y., Reis e Sousa C. (2006). Nucleic acid agonists for Toll-like receptor 7 are defined by the presence of uridine ribonucleotides. Eur. J. Immunol..

[64] Gorden K.K.B., Qiu X., Battiste J.J.L., Wightman P.P.D., Vasilakos J.P., Alkan S.S. (2006). Oligodeoxynucleotides Differentially Modulate Activation of TLR7 and TLR8 by Imidazoquinolines. J. Immunol..

[65] Karikó K., Buckstein M., Ni H., Weissman D. (2005). Suppression of RNA recognition by Toll-like receptors: The impact of nucleoside modification and the evolutionary origin of RNA. Immunity.

[66] Lorenz C., Fotin-Mleczek M., Roth G., Becker C., Dam T.C., Verdurmen W.P.R., Brock R., Probst J., Schlake T. (2011). Protein expression from exogenous mRNA: Uptake by receptor-mediated endocytosis and trafficking via the lysosomal pathway. RNA Biol..

[67] Svitkin Y.V., Cheng Y.M., Chakraborty T., Presnyak V., John M., Sonenberg N. (2017). N1-methyl-pseudouridine in mRNA enhances translation through eIF2α-dependent and independent mechanisms by increasing ribosome density. Nucleic Acids Res..

[68] Andries O., Mc Cafferty S., De Smedt S.C., Weiss R., Sanders N.N., Kitada T. (2015). N1-methylpseudouridine-incorporated mRNA outperforms pseudouridine-incorporated mRNA by providing enhanced protein expression and reduced immunogenicity in mammalian cell lines and mice. J. Control. Release.

[69] Martini P.G.V., Guey L.T. (2019). A New Era for Rare Genetic Diseases: Messenger RNA Therapy. Hum. Gene Ther..

[70] Meyer K.D., Patil D.P., Zhou J., Zinoviev A., Skabkin M.A., Elemento O., Pestova T.V., Qian S.-B., Jaffrey S.R. (2015). 5′ UTR m6A Promotes Cap-Independent Translation. Cell.

[71] Ulkoski D., Bak A., Wilson J.T., Krishnamurthy V.R. (2019). Recent advances in polymeric materials for the delivery of RNA therapeutics. Expert Opin. Drug Deliv..

[72] Rodríguez-Gascón A., del Pozo-Rodríguez A., Solinís M.Á. (2014). Development of nucleic acid vaccines: Use of self-amplifying RNA in lipid nanoparticles. Int. J. Nanomed..

[73] (2019). Gene Therapy Clinical Trials Worldwide, Provided by the Journal of Gene Medicine, Jon Wiley and Sons Ltd. http://www.abedia.com/wiley/vectors.php.

[74] Carvalho M., Sepodes B., Martins A.P. (2017). Regulatory and Scientific Advancements in Gene Therapy: State-of-the-Art of Clinical Applications and of the Supporting European Regulatory Framework. Front. Med..

[75] del Pozo-Rodríguez A., Solinís M.Á., Rodríguez-Gascón A. (2016). Applications of lipid nanoparticles in gene therapy. Eur. J. Pharm. Biopharm..

[76] Wang Y., Rajala A., Rajala R. (2015). Lipid Nanoparticles for Ocular Gene Delivery. J. Funct. Biomater..

[77] Yin H., Kanasty R.L., Eltoukhy A.A., Vegas A.J., Dorkin J.R., Anderson D.G. (2014). Non-viral vectors for gene-based therapy. Nat. Rev. Genet..

[78] Guan S., Rosenecker J. (2017). Nanotechnologies in delivery of mRNA therapeutics using nonviral vector-based delivery systems. Gene Ther..

[79] Rodríguez-Gascón A., del Pozo-Rodríguez A., Isla A., Solinís M.A. (2015). Vaginal gene therapy. Adv. Drug Deliv. Rev..

[80] Gascón A.R., del Pozo-Rodríguez A., Solinís M.A., Molina F.M. (2013). Non-Viral Delivery Systems in Gene Therapy. Gene Therapy.

[81] Van Tendeloo V.F.I., Ponsaerts P., Lardon F., Nijs G., Lenjou M., Van Broeckhoven C., Van Bockstaele D.R.V., Berneman Z.N. (2001). Highly efficient gene delivery by mRNA electroporation in human hematopoietic cells: Superiority to lipofection and passive pulsing of mRNA and to electroporation of plasmid cDNA for tumor antigen loading of dendritic cells. Blood.

[82] Bugeon S., De Chevigny A., Boutin C., Coré N., Wild S., Bosio A. (2017). Harold Cremer 3, Christophe Beclin Direct and efficient transfection of mouse neural stem cells and mature neurons by in vivo mRNA electroporation. Development.

[83] Golombek S., Pilz M., Steinle H., Kochba E., Levin Y., Lunter D., Schlensak C., Wendel H.P., Avci-Adali M. (2018). Intradermal Delivery of Synthetic mRNA Using Hollow Microneedles for Efficient and Rapid Production of Exogenous Proteins in Skin. Mol. Ther Nucleic Acids.

[84] Moody S.A. (2018). Microinjection of mRNAs and oligonucleotides. Cold Spring Harb. Protoc..

[85] Ainger K., Avossa D., Morgan F., Hill S.J., Barry C., Barbarese E., Carson J.H. (1993). Transport and localization of exogenous myelin basic protein mRNA microinjected into oligodendrocytes. J. Cell Biol..

[86] Belyantseva I.A., Sokolowski B. (2016). Helios® Gene Gun-Mediated Transfection of the Inner Ear Sensory Epithelium: Recent Updates. Auditory and Vestibular Research: Methods and Protocols.

[87] Vassilev V.B., Gil L.H.V.G., Donis R.O. (2001). Microparticle-mediated RNA immunization against bovine viral diarrhea virus. Vaccine.

[88] Ramezanpour M., Schmidt M.L., Bodnariuc I., Kulkarni J.A., Leung S.S.W., Cullis P.R., Thewalt J.L., Tieleman D.P. (2019). Ionizable amino lipid interactions with POPC: Implications for lipid nanoparticle function. Nanoscale.

[89] Islam M.A., Xu Y., Tao W., Ubellacker J.M., Lim M., Aum D., Lee G.Y., Zhou K., Zope H., Yu M. (2018). Restoration of tumour-growth suppression in vivo via systemic nanoparticle-mediated delivery of PTEN mRNA. Nat. Biomed. Eng..

[90] Kauffman K.J., Webber M.J., Anderson D.G. (2016). Materials for non-viral intracellular delivery of messenger RNA therapeutics. J. Control. Release.

[91] Phua K.K.L., Leong K.W., Nair S.K. (2013). Transfection Efficiency and Transgene Expression Kinetics of mRNA Delivered in Naked and Nanoparticle Format. J. Control. Release.

[92] Slivac I., Guay D., Mangion M., Champeil J., Gaillet B. (2017). Non-viral nucleic acid delivery methods. Expert Opin. Biol. Ther..

[93] Aderibigbe B.A., Ray S.S. (2016). Preparation, characterization and in vitro release kinetics of polyaspartamide-based conjugates containing antimalarial and anticancer agents for combination therapy. J. Drug Deliv. Sci. Technol..

[94] Malone R.W., Felgner P.L., Verma I.M. (1989). Cationic liposome-mediated RNA transfection. Proc. Natl. Acad. Sci. USA.

[95] Koltover I., Salditt T., Rädler J.O., Safinya C.R. (1998). An inverted hexagonal phase of cationic liposome-DNA complexes related to DNA release and delivery. Science.

[96] Sayour E.J., De Leon G., Pham C., Grippin A., Kemeny H., Chua J., Huang J., Sampson J.H., Sanchez-Perez L., Flores C. (2017). Systemic activation of antigen-presenting cells via RNA-Loaded nanoparticles. Oncoimmunology.

[97] Kranz L.M., Diken M., Haas H., Kreiter S., Loquai C., Reuter K.C., Meng M., Fritz D., Fulvia V., Hefesha H. (2016). Systemic RNA delivery to dendritic cells exploits antiviral defence for cancer immunotherapy. Nature.

[98] Kulkarni J.A., Cullis P.R., Van Der Meel R. (2018). Lipid Nanoparticles Enabling Gene Therapies: From Concepts to Clinical Utility. Nucleic Acid Ther..

[99] Bogers W.M., Oostermeijer H., Mooij P., Koopman G., Verschoor E.J., Davis D., Ulmer J.B., Brito L.A., Cu Y., Banerjee K. (2015). Potent immune responses in rhesus macaques induced by nonviral delivery of a self-amplifying RNA vaccine expressing HIV type 1 envelope with a cationic nanoemulsion. J. Infect. Dis..

[100] Brito L.A., Chan M., Shaw C.A., Hekele A., Carsillo T., Schaefer M., Archer J., Seubert A., Otten G.R., Beard C.W. (2014). A cationic nanoemulsion for the delivery of next-generation RNA vaccines. Mol. Ther..

[101] Kauffman K.J., Dorkin J.R., Yang J.H., Heartlein M.W., Derosa F., Mir F.F., Fenton O.S., Anderson D.G. (2015). Optimization of Lipid Nanoparticle Formulations for mRNA Delivery in Vivo with Fractional Factorial and Definitive Screening Designs. Nano Lett..

[102] Adams D., Gonzalez-Duarte A., O’Riordan W.D., Yang C.C., Ueda M., Kristen A.V., Tournev I., Schmidt H.H., Coelho T., Berk J.L. (2018). Patisiran, an RNAi therapeutic, for hereditary transthyretin amyloidosis. N. Engl. J. Med..

[103] Zhang X., Goel V., Robbie G.J. (2019). Pharmacokinetics of Patisiran, the First Approved RNA Interference Therapy in Patients with Hereditary Transthyretin-Mediated Amyloidosis. J. Clin. Pharmacol..

[104] Sedic M., Senn J.J., Lynn A., Laska M., Smith M., Platz S.J., Bolen J., Hoge S., Bulychev A., Jacquinet E. (2018). Safety Evaluation of Lipid Nanoparticle–Formulated Modified mRNA in the Sprague-Dawley Rat and Cynomolgus Monkey. Vet. Pathol..

[105] Lu D., Benjamin R., Kim M., Conry R.M., Curiel D.T. (1994). Optimization of methods to achieve mRNA-mediated transfection of tumor cells in vitro and in vivo employing cationic liposome vectors. Cancer Gene Ther..

[106] Pardi N., Hogan M.J., Pelc R.S., Muramatsu H., Andersen H., DeMaso C.R., Dowd K.A., Sutherland L.L., Scearce R.M., Parks R. (2017). Zika virus protection by a single low-dose nucleoside-modified mRNA vaccination. Nature.

[107] Hekele A., Bertholet S., Archer J., Gibson D.G., Palladino G., Brito L.A., Hekele A., Bertholet S., Archer J., Gibson D.G. (2013). Rapidly produced SAM vaccine against H7N9 influenza is immunogenic in mice. Emerg. Microbes Infect..

[108] Zhou W.Z., Hoon D.S.B., Huang S.K.S., Fujii S., Hashimoto K., Morishita R., Kaneda Y. (1999). RNA melanoma vaccine: Induction of antitumor immunity by human glycoprotein 100 mRNA immunization. Hum. Gene. Ther..

[109] Finn J.D., Smith A.R., Patel M.C., Shaw L., Youniss M.R., van Heteren J., Dirstine T., Ciullo C., Lescarbeau R., Seitzer J. (2018). A Single Administration of CRISPR/Cas9 Lipid Nanoparticles Achieves Robust and Persistent In Vivo Genome Editing. Cell Rep..

[110] Kormann M.S.D., Hasenpusch G., Aneja M.K., Nica G., Flemmer A.W., Herber-Jonat S., Huppmann M., Mays L.E., Illenyi M., Schams A. (2011). Expression of therapeutic proteins after delivery of chemically modified mRNA in mice. Nat. Biotechnol..

[111] Akinc A., Zumbuehl A., Goldberg M., Leshchiner E.S., Busini V., Hossain N., Bacallado S.A., Nguyen D.N., Fuller J., Alvarez R. (2008). A combinatorial library of lipid-like materials for delivery of RNAi therapeutics. Nat. Biotechnol..

[112] Miller J.B., Zhang S., Kos P., Xiong H., Zhou K., Perelman S.S., Zhu H., Siegwart D.J. (2017). Non-Viral CRISPR/Cas Gene Editing In Vitro and In Vivo Enabled by Synthetic Nanoparticle Co-Delivery of Cas9 mRNA and sgRNA. Angew. Chem. Int. Ed..

[113] Li B., Luo X., Deng B., Wang J., McComb D.W., Shi Y., Gaensler K.M.L., Tan X., Dunn A.L., Kerlin B.A. (2015). An Orthogonal Array Optimization of Lipid-like Nanoparticles for mRNA Delivery in Vivo. Nano Lett..

[114] Nagpal G., Chaudhary K., Dhanda S.K., Pal G., Raghava S. (2017). Computational Prediction of the Immunomodulatory Potential of RNA Sequences. Methods Mol. Biol..

[115] Beloqui A., Solinís M.Á., Rodríguez-Gascón A., Almeida A.J., Préat V. (2016). Nanostructured lipid carriers: Promising drug delivery systems for future clinics. Nanomed. Nanotechnol. Biol. Med..

[116] Beloqui A., del Pozo-Rodríguez A., Isla A., Rodríguez-Gascón A., Solinís M.Á. (2017). Nanostructured lipid carriers as oral delivery systems for poorly soluble drugs. J. Drug Deliv. Sci. Technol..

[117] Beloqui A., Solinís M.Á., des Rieux A., Préat V., Rodríguez-Gascón A. (2014). Dextran–protamine coated nanostructured lipid carriers as mucus-penetrating nanoparticles for lipophilic drugs. Int. J. Pharm..

[118] Erasmus J.H., Khandhar A.P., Guderian J., Granger B., Archer J., Archer M., Gage E., Fuerte-Stone J., Larson E., Lin S. (2018). A Nanostructured Lipid Carrier for Delivery of a Replicating Viral RNA Provides Single, Low-Dose Protection against Zika. Mol. Ther..

[119] Godbey W.T., Wu K.K., Mikos A.G. (1999). Size matters: Molecular weight affects the efficiency of poly(ethylenimine) as a gene delivery vehicle. J. Biomed. Mater. Res..

[120] Lv H., Zhang S., Wang B., Cui S., Yan J. (2006). Toxicity of cationic lipids and cationic polymers in gene delivery. J. Control. Release.

[121] Rejman J., Tavernier G., Bavarsad N., Demeester J., De Smedt S.C. (2010). MRNA transfection of cervical carcinoma and mesenchymal stem cells mediated by cationic carriers. J. Control. Release.

[122] Sultana N., Magadum A., Hadas Y., Kondrat J., Singh N., Youssef E., Calderon D., Chepurko E., Dubois N., Hajjar R.J. (2017). Optimizing Cardiac Delivery of Modified mRNA. Mol. Ther..

[123] Üzgün S., Nica G., Pfeifer C., Bosinco M., Michaelis K., Lutz J.F., Schneider M., Rosenecker J., Rudolph C. (2011). PEGylation improves nanoparticle formation and transfection efficiency of messenger RNA. Pharm. Res..

[124] Cheng C., Convertine A.J., Stayton P.S., Bryers J.D. (2012). Multifunctional triblock copolymers for intracellular messenger RNA delivery. Biomaterials.

[125] Jarzębińska A., Pasewald T., Lambrecht J., Mykhaylyk O., Kümmerling L., Beck P., Hasenpusch G., Rudolph C., Plank C., Dohmen C. (2016). A Single Methylene Group in Oligoalkylamine-Based Cationic Polymers and Lipids Promotes Enhanced mRNA Delivery. Angew. Chem. Int. Ed..

[126] Liu Y., Li Y., Keskin D., Shi L. (2019). Poly (β-Amino Esters): Synthesis, Formulations, and Their Biomedical Applications. Adv. Healthc. Mater..

[127] Kaczmarek J.C., Patel A.K., Kauffman K.J., Fenton O.S., Webber M.J., Heartlein M.W., DeRosa F. (2016). Daniel G Anderson. Polymer–Lipid Nanoparticles for Systemic Delivery of mRNA to the Lungs. Angew. Chem. Int. Ed..

[128] Patel A.K., Kaczmarek J.C., Bose S., Kauffman K.J., Mir F., Heartlein M.W., DeRosa F., Langer R., Anderson D.G. (2019). Inhaled Nanoformulated mRNA Polyplexes for Protein Production in Lung Epithelium. Adv. Mater..

[129] Uchida S., Kataoka K. (2019). Design concepts of polyplex micelles for in vivo therapeutic delivery of plasmid DNA and messenger RNA. J. Biomed. Mater. Res. Part A.

[130] Uchida H., Itaka K., Nomoto T., Ishii T., Suma T., Ikegami M., Miyata K., Oba M., Nishiyama N., Kataoka K. (2014). Modulated protonation of side chain aminoethylene repeats in N-substituted polyaspartamides promotes mRNA transfection. J. Am. Chem. Soc..

[131] Baba M., Itaka K., Kondo K., Yamasoba T., Kataoka K. (2015). Treatment of neurological disorders by introducing mRNA in vivo using polyplex nanomicelles. J. Control. Release.

[132] Matsui A., Uchida S., Ishii T., Itaka K., Kataoka K. (2015). Messenger RNA-based therapeutics for the treatment of apoptosis-associated diseases. Sci. Rep..

[133] Palamà I.E., Cortese B., D’Amone S., Gigli G. (2015). MRNA delivery using non-viral PCL nanoparticles. Biomater. Sci..

[134] Lallana E., Rios de la Rosa J.M., Tirella A., Pelliccia M., Gennari A., Stratford I.J., Puri S., Ashford M., Tirelli N. (2017). Chitosan/Hyaluronic Acid Nanoparticles: Rational Design Revisited for RNA Delivery. Mol. Pharm..

[135] McKinlay C.J., Vargas J.R., Blake T.R., Hardy J.W., Kanada M., Contag C.H., Wender P.A., Waymouth R.M. (2017). Charge-altering releasable transporters (CARTs) for the delivery and release of mRNA in living animals. Proc. Natl. Acad. Sci. USA.

[136] McKinlay C.J., Benner N.L., Haabeth O.A., Waymouth R.M., Wender P.A. (2018). Enhanced mRNA delivery into lymphocytes enabled by lipid-varied libraries of charge-altering releasable transporters. Proc. Natl. Acad. Sci. USA.

[137] Haabeth O.A.W., Blake T.R., McKinlay C.J., Waymouth R.M., Wender P.A., Levy R. (2018). mRNA vaccination with charge-altering releasable transporters elicits human T cell responses and cures established tumors in mice. Proc. Natl. Acad. Sci. USA.

[138] Armbruster N., Jasny E., Petsch B. (2019). Advances in RNA Vaccines for Preventive Indications: A Case Study of A Vaccine Against Rabies. Vaccines.

[139] Amos H. (1961). Protamine enhancement of RNA uptake by cultured chick cells. Biochem. Biophys. Res. Commun..

[140] Scheel B., Teufel R., Probst J., Carralot J.P., Geginat J., Radsak M., Jarrossay D., Wagner H., Jung G., Rammensee H.-G. (2005). Toll-like receptor-dependent activation of several human blood cell types by protamine-condensed mRNA. Eur. J. Immunol..

[141] Sebastian M., Schröder A., Scheel B., Hong H.S., Muth A., von Boehmer L., Zippelius A., Mayer F., Reck M., Atanackovic D. (2019). A phase I/IIa study of the mRNA-based cancer immunotherapy CV9201 in patients with stage IIIB/IV non-small cell lung cancer. Cancer Immunol. Immunother..

[142] Kübler H., Scheel B., Gnad-Vogt U., Miller K., Schultze-Seemann W., Dorp F., Parmiani G., Hampel C., Wedel S., Trojan L. (2015). Self-adjuvanted mRNA vaccination in advanced prostate cancer patients: A first-in-man phase I/IIa study. J. Immunother. Cancer.

[143] Petsch B., Schnee M., Vogel A.B., Lange E., Hoffmann B., Voss D., Schlake T., Thess A., Kallen K.J., Stitz L. (2012). Protective efficacy of in vitro synthesized, specific mRNA vaccines against influenza A virus infection. Nat. Biotechnol..

[144] Udhayakumar V.K., De Beuckelaer A., McCaffrey J., McCrudden C.M., Kirschman J.L., Vanover D., Hoecke L.V., Roose K., Deswarte K., De Geest B.G. (2017). Arginine-Rich Peptide-Based mRNA Nanocomplexes Efficiently Instigate Cytotoxic T Cell Immunity Dependent on the Amphipathic Organization of the Peptide. Adv. Healthc. Mater..

[145] Li J., Sun Y., Jia T., Zhang R., Zhang K., Wang L. (2014). Messenger RNA vaccine based on recombinant MS2 virus-like particles against prostate cancer. Int. J. Cancer.

[146] Jekhmane S., de Haas R., da Silva Filho O., van Asbeck A.H., Favretto M.E., Hernandez Garcia A., Brock R. (2017). Renko de Vries Virus-Like Particles of mRNA with Artificial Minimal Coat Proteins: Particle Formation, Stability, and Transfection Efficiency. Nucleic Acid Ther..

[147] Zhitnyuk Y., Gee P., Lung M.S.Y., Sasakawa N., Xu H., Saito H., Hotta A. (2018). Efficient mRNA delivery system utilizing chimeric VSVG-L7Ae virus-like particles. Biochem. Biophys. Res. Commun..

[148] Palmerston Mendes L., Pan J., Torchilin V.P. (2017). Dendrimers as Nanocarriers for Nucleic Acid and Drug Delivery in Cancer Therapy. Molecules.

[149] Chahal J.S., Khan O.F., Cooper C.L., McPartlan J.S., Tsosie J.K., Tilley L.D., Sidik S.M., Lourido S., Langer R., Bavari S. (2016). Dendrimer-RNA nanoparticles generate protective immunity against lethal Ebola, H1N1 influenza, and Toxoplasma gondii challenges with a single dose. Proc. Natl. Acad. Sci. USA.

[150] Chahal J.S., Fang T., Woodham A.W., Khan O.F., Ling J., Anderson D.G., Ploegh H.L. (2017). An RNA nanoparticle vaccine against Zika virus elicits antibody and CD8+ T cell responses in a mouse model. Sci. Rep..

[151] Ding Y., Jiang Z., Saha K., Kim C.S., Kim S.T., Landis R.F., Rotello V.M. (2014). Gold Nanoparticles for Nucleic Acid Delivery. Mol. Ther..

[152] Yeom J.H., Ryou S.M., Won M., Park M., Bae J., Lee K. (2013). Inhibition of Xenograft Tumor Growth by Gold Nanoparticle-DNA Oligonucleotide Conjugates-Assisted Delivery of BAX mRNA. PLoS ONE.

[153] Hoerr I., Obst R., Rammensee H.G., Jung G. (2000). In vivo application of RNA leads to induction of specific cytotoxic T lymphocytes and antibodies. Eur. J. Immunol..

[154] Wang Y., Su H.H., Yang Y., Hu Y., Zhang L., Blancafort P., Huang L. (2013). Systemic delivery of modified mRNA encoding herpes simplex virus 1 thymidine kinase for targeted cancer gene therapy. Mol. Ther..

[155] Lee K., Yu P., Lingampalli N., Kim H.J., Tang R., Murthy N. (2015). Peptide-enhanced mRNA transfection in cultured mouse cardiac fibroblasts and direct reprogramming towards cardiomyocyte-like cells. Int. J. Nanomed..

[156] Zohra F.T., Chowdhury E.H., Akaike T. (2009). High performance mRNA transfection through carbonate apatite-cationic liposome conjugates. Biomaterials.

[157] Mockey M., Bourseau E., Chandrashekhar V., Chaudhuri A., Lafosse S., Le Cam E., Quesniaux V.F., Ryffel B., Pichon C., Midoux P. (2007). mRNA-based cancer vaccine: Prevention of B16 melanoma progression and metastasis by systemic injection of MART1 mRNA histidylated lipopolyplexes. Cancer Gene Ther..

[158] Pichon C., Midoux P., Rabinovich P.M. (2013). Mannosylated and Histidylated LPR Technology for Vaccination with Tumor Antigen mRNA. Synthetic Messenger RNA and Cell Metabolism Modulation: Methods and Protocols.

[159] Uchida S., Kinoh H., Ishii T., Matsui A., Tockary T.A., Takeda K.M., Uchida H., Osada K., Itaka K., Kataoka K. (2016). Systemic delivery of messenger RNA for the treatment of pancreatic cancer using polyplex nanomicelles with a cholesterol moiety. Biomaterials.

[160] Dong Y., Dorkin J.R., Wang W., Chang P.H., Webber M.J., Tang B.C., Yang J., Abutbul-lonita I., Danino D., DeRosa F. (2016). Poly (glycoamidoamine) Brushes Formulated Nanomaterials for Systemic siRNA and mRNA Delivery in Vivo. Nano Lett..

[161] Lacroix C., Humanes A., Coiffier C., Gigmes D., Verrier B., Trimaille T. (2020). Polylactide-Based Reactive Micelles as a Robust Platform for mRNA Delivery. Pharm. Res..

[162] Fishman S., Lewis M.D., Siew L.K., Leenheer EDe Kakabadse D., Davies J., Ziv D., Margalit A., Karin N., Gross G. (2017). Adoptive Transfer of mRNA-Transfected T Cells Redirected against Diabetogenic CD8 T Cells Can Prevent Diabetes. Mol. Ther..

[163] Van Hoecke L., Roose K. (2019). How mRNA therapeutics are entering the monoclonal antibody field. J. Transl. Med..

[164] Pardi N., Hogan M.J., Porter F.W., Weissman D. (2018). mRNA vaccines-a new era in vaccinology. Nat. Rev. Drug Discov..

[165] Liu A.M. (2019). A Comparison of Plasmid DNA and mRNA as Vaccine Technologies. Vaccines.

[166] Maruggi G., Zhang C., Li J., Ulmer J.B., Yu D. (2019). mRNA as a Transformative Technology for Vaccine Development to Control Infectious Diseases. Mol. Ther..

[167] Zhou X., Berglund P., Rhodes G., Parker S.E., Jondal M., Liljeström P. (1994). Self-replicating Semliki Forest virus RNA as recombinant vaccine. Vaccine.

[168] Fleeton M.N., Chen M., Berglund P., Rhodes G., Parker S.E., Murphy M., Atkins G.J., Liljeström P. (2001). Self-Replicative RNA Vaccines Elicit Protection against Influenza A Virus, Respiratory Syncytial Virus, and a Tickborne Encephalitis Virus. J. Infect. Dis..

[169] Geall A.J., Verma A., Otten G.R., Shaw C.A., Hekele A., Banerjee K., Cu Y., Beard C.W., Brito L.A., Krucker T. (2012). Nonviral delivery of self-amplifying RNA vaccines. Proc. Natl. Acad. Sci. USA.

[170] Bernstein D.I., Reap E.A., Katen K., Watson A., Smith K., Norberg P., Olmsted R.A., Hoeper A., Morris J., Negri S. (2009). Randomized, double-blind, Phase 1 trial of an alphavirus replicon vaccine for cytomegalovirus in CMV seronegative adult volunteers. Vaccine.

[171] AlphaVax. https://www.alphavax.com/clinical-experience.html.

[172] Wecker M., Gilbert P., Russell N., Hural J., Allen M., Pensiero M., Chulay J., Chiu Y.L., Abdool Karim S.S., Burke D.S. (2012). Phase I Safety and Immunogenicity Evaluations of an Alphavirus Replicon HIV-1 Subtype C gag vaccine in healthy HIV-1-uninfected adults. Clin. Vaccine Immunol..

[173] Schnee M., Vogel A.B., Voss D., Petsch B., Baumhof P., Kramps T., Stitz L. (2016). An mRNA Vaccine Encoding Rabies Virus Glycoprotein Induces Protection against Lethal Infection in Mice and Correlates of Protection in Adult and Newborn Pigs. PLoS Negl. Trop. Dis..

[174] Alberer M., Gnad-Vogt U., Hong H.S., Mehr K.T., Backert L., Finak G., Gottardo R., Bica M.A., Garofano A., Koch S.D. (2017). Safety and immunogenicity of a mRNA rabies vaccine in healthy adults: An open-label, non-randomised, prospective, first-in-human phase 1 clinical trial. Lancet.

[175] Richner J.M., Himansu S., Dowd K.A., Butler S.L., Salazar V., Fox J.M., Julander J.G., Tang W.W., Shresta S., Pierson T.C. (2017). Modified mRNA Vaccines Protect against Zika Virus Infection. Cell.

[176] Bahl K., Senn J.J., Yuzhakov O., Bulychev A., Brito L.A., Hassett K.J., Laska M.E., Smith M., Almarsson Ö., Thompson J. (2017). Preclinical and Clinical Demonstration of Immunogenicity by mRNA Vaccines against H10N8 and H7N9 Influenza Viruses. Mol. Ther..

[177] Feldman R.A., Fuhr R., Smolenov I., (Mick) Ribeiro A., Panther L., Watson M., Senn J.J., Smith M., Almarsson ö., Pujar H.S. (2019). mRNA vaccines against H10N8 and H7N9 influenza viruses of pandemic potential are immunogenic and well tolerated in healthy adults in phase 1 randomized clinical trials. Vaccine.

[178] Leal L., Guardo A.C., Morón-López S., Salgado M., Mothe B., Heirman C., Pannus P., Vanham G., van den Ham H.J., Gruters R. (2018). Phase I clinical trial of an intranodally administered mRNA-based therapeutic vaccine against HIV-1 infection. AIDS.

[179] Van Lint S., Renmans D., Broos K., Goethals L., Maenhout S., Benteyn D., Goyvaerts C., Du Four S., Van der Jeught K., Bialkowski L. (2016). Intratumoral Delivery of TriMix mRNA Results in T-cell Activation by Cross-Presenting Dendritic Cells. Cancer Immunol. Res..

[180] Gandhi R.T., Kwon D.S., Macklin E.A., Shopis J.R., McLean A.P., McBrine N., Flynn T., Peter L., Sbrolla A., Kaufmann D.E. (2016). Immunization of HIV-1-Infected Persons with Autologous Dendritic Cells Transfected With mRNA Encoding HIV-1 Gag and Nef: Results of a Randomized, Placebo-Controlled Clinical Trial. J. Acquir. Immune Defic. Syndr..

[181] McNamara M.A., Nair S.K., Holl E.K. (2015). RNA-Based Vaccines in Cancer Immunotherapy. J. Immunol. Res..

[182] Kavanagh D.G., Kaufmann D.E., Sunderji S., Frahm N., Le Gall S., Boczkowski D., Rosenberg E.S., Stone D.R., Johnston M.N., Wagner B.S. (2006). Expansion of HIV-specific CD4+ and CD8+ T cells by dendritic cells transfected with mRNA encoding cytoplasm- or lysosome-targeted Nef. Blood.

[183] Melhem N.M., Liu X.D., Boczkowski D., Gilboa E., Barratt-Boyes S.M. (2007). Robust CD4+ and CD8+ T cell responses to SIV using mRNA-transfected DC expressing autologous viral Ag. Eur. J. Immunol..

[184] Van Nuffel A.M.T., Benteyn D., Wilgenhof S., Pierret L., Corthals J., Heirman C., van der Bruggen P., Coulie P.G., Neyns B., Thielemans K. (2012). Dendritic Cells Loaded with mRNA Encoding Full-length Tumor Antigens Prime CD4+ and CD8+ T Cells in Melanoma Patients. Mol. Ther..

[185] Eisenächer K., Steinberg C., Reindl W., Krug A. (2007). The role of viral nucleic acid recognition in dendritic cells for innate and adaptive antiviral immunity. Immunobiology.

[186] Wirth T.C., Kühnel F. (2017). Neoantigen Targeting—Dawn of a New Era in Cancer Immunotherapy?. Front. Immunol..

[187] Sahin U., Derhovanessian E., Miller M., Kloke B.P., Simon P., Löwer M., Bukur V., Tadmor A.D., Luxemburger U., Schrörs B. (2017). Personalized RNA mutanome vaccines mobilize poly-specific therapeutic immunity against cancer. Nature.

[188] Conry R.M., LoBuglio A.F., Wright M., Sumerel L., Pike M.J., Johanning F., Benjamis R., Lu D., Curiel D.T. (1995). Characterization of a Messenger RNA Polynucleotide Vaccine Vector. Cancer Res..

[189] Papachristofilou A., Hipp M.M., Klinkhardt U., Früh M., Sebastian M., Weiss C., Pless M., Cathomas R., Hilbe W., Pall G. (2019). Phase Ib evaluation of a self-adjuvanted protamine formulated mRNA-based active cancer immunotherapy, BI1361849 (CV9202), combined with local radiation treatment in patients with stage IV non-small cell lung cancer. J. Immunother. Cancer.

[190] Boczkowski B.D., Nair S.K., Snyder D., Gilboa E. (1996). Dendritic Cells Pulsed with RNA are Potent Antigen-presenting Cells In Vitro and In Vivo. J. Exp. Med..

[191] Kyte J.A., Mu L., Aamdal S., Kvalheim G., Dueland S., Hauser M., Gullestad H.P., Ryder T., Lislerud K., Hammerstad H. (2006). Phase I/II trial of melanoma therapy with dendritic cells transfected with autologous tumor-mRNA. Cancer Gene Ther..

[192] Wilgenhof S., Van Nuffel A.M.T., Benteyn D., Corthals J., Aerts C., Heirman C., Van Riet I., Bonehill A., Thielemans K., Neyns B. (2013). A phase IB study on intravenous synthetic mRNA electroporated dendritic cell immunotherapy in pretreated advanced melanoma patients. Ann. Oncol..

[193] Wilgenhof S., Corthals J., Heirman C., Van Baren N., Lucas S., Kvistborg P., Thielemans K., Neyns B. (2016). Phase II study of autologous monocyte-derived mRNA electroporated dendritic cells (TriMixDC-MEL) plus ipilimumab in patientswith pretreated advanced melanoma. J. Clin. Oncol..

[194] Nogrady B. (2018). Gene therapy delivers hope. Nature.

[195] Rabinovich P.M., Komarovskaya M.E., Ye Z.J., Imai C., Campana D., Bahceci E., Weissman S.M. (2006). Synthetic messenger RNA as a tool for gene therapy. Hum. Gene Ther..

[196] Foster J.B., Barrett D.M., Karikó K. (2019). The Emerging Role of In Vitro-Transcribed mRNA in Adoptive T Cell Immunotherapy. Mol. Ther..

[197] Maus M.V., Haas A.R., Beatty G.L., Albelda S.M., Levine B.L., Liu X., Zhao Y., Kalos M., June C.H. (2013). T Cells Expressing Chimeric Antigen Receptors Can Cause Anaphylaxis in Humans. Cancer Immunol. Res..

[198] Svoboda J., Rheingold S.R., Gill S.I., Grupp S.A., Lacey S.F., Kulikovskaya I., Suhoski M.M., Melenhorst J.J., Loudon B., Mato A.R. (2018). Nonviral RNA chimeric antigen receptor–modified T cells in patients with Hodgkin lymphoma. Blood.

[199] Tchou J., Zhao Y., Levine B.L., Zhang P.J., Davis M.M., Melenhorst J.J., Kulikovskaya I., Brennan A.L., Liu X., Lacey S.F. (2017). Safety and Efficacy of Intratumoral Injections of Chimeric Antigen Receptor (CAR) T Cells in Metastatic Breast Cancer. Cancer Immunol. Res..

[200] Jirikowski G.F., Sanna P.P., Maciejewski-Lenoir D., Bloom F.E. (1992). Reversal of diabetes insipidus in Brattleboro tats: Intrahypothalamic injection of vasopressin mRNA. Science.

[201] Trepotec Z., Lichtenegger E., Plank C., Aneja M.K., Rudolph C. (2019). Delivery of mRNA Therapeutics for the Treatment of Hepatic Diseases. Mol. Ther..

[202] Sahu I., Haque A.K.M.A., Weidensee B., Weinmann P., Kormann M.S.D. (2019). Recent Developments in mRNA-Based Protein Supplementation Therapy to Target Lung Diseases. Mol. Ther..

[203] Magadum A., Kaur K., Zangi L. (2019). mRNA-Based Protein Replacement Therapy for the Heart. Mol. Ther..

[204] Lescan M., Perl R.M., Golombek S., Pilz M., Hann L., Yasmin M., Behring A., Keller T., Nolte A., Gruhn F. (2018). De Novo Synthesis of Elastin by Exogenous Delivery of Synthetic Modified mRNA into Skin and Elastin-Deficient Cells. Mol. Ther. Nucleic Acids.

[205] Patel S., Ryals R.C., Weller K.K., Pennesi M.E., Sahay G. (2019). Lipid nanoparticles for delivery of messenger RNA to the back of the eye. J. Control. Release.

[206] DeRosa F., Guild B., Karve S., Smith L., Love K., Dorkin J.R., Kauffman K.J., Zhang J., Yahalom B., Anderson D.G. (2016). Therapeutic efficacy in a hemophilia B model using a biosynthetic mRNA liver depot system. Gene Ther..

[207] Zhu X., Yin L., Theisen M., Zhuo J., Siddiqui S., Levy B., Presnyak V., Frassetto A., Milton J., Salerno T. (2019). Systemic mRNA Therapy for the Treatment of Fabry Disease: Preclinical Studies in Wild-Type Mice, Fabry Mouse Model, and Wild-Type Non-human Primates. Am. J. Hum. Genet..

[208] An D., Schneller J.L., Frassetto A., Liang S., Zhu X., Park J.S., Theisen M., Hong S.J., Zhou J., Rajendran R. (2017). Systemic Messenger RNA Therapy as a Treatment for Methylmalonic Acidemia. Cell Rep..

[209] Moderna. https://investors.modernatx.com/news-releases/news-release-details/moderna-announces-open-ind-propionic-acidemia-program-mrna-3927/.

[210] Jiang L., Berraondo P., Jericó D., Guey L.T., Sampedro A., Frassetto A., Benenato K.E., Burke K., Santamaría E., Alegre M. (2018). Systemic messenger RNA as an etiological treatment for acute intermittent porphyria. Nat. Med..

[211] Berraondo P., Martini P.G.V., Avila M.A., Fontanellas A. (2019). Messenger RNA therapy for rare genetic metabolic diseases. Gut.

[212] Robinson E., MacDonald K.D., Slaughter K., McKinney M., Patel S., Sun C., Sahay G. (2018). Lipid Nanoparticle-Delivered Chemically Modified mRNA Restores Chloride Secretion in Cystic Fibrosis. Mol. Ther..

[213] Carlsson L., Clarke J.C., Yen C., Gregoire F., Albery T., Billger M., Egnell A.C., Gan L.M., Jennbacken K., Johansson E. (2018). Biocompatible, Purified VEGF-A mRNA Improves Cardiac Function after Intracardiac Injection 1 Week Post-myocardial Infarction in Swine. Mol. Ther. Methods Clin. Dev..

[214] Gan L.-M., Lagerström-Fermér M., Carlsson L.G., Arfvidsson C., Egnell A.-C., Rudvik A., Kjaer M., Collén A., Thompson J.D., Joyal J. (2019). Intradermal delivery of modified mRNA encoding VEGF-A in patients with type 2 diabetes. Nat. Commun..

[215] Genetics Home reference. https://ghr.nlm.nih.gov/condition/propionic-acidemia.

[216] Fraser J.L., Venditti C.P. (2016). Methylmalonic and propionic acidemias: Clinical management update. Curr. Opin. Pediatr..

[217] Critelli K., McKiernan P., Vockley J., Mazariegos G., Squires R.H., Soltys K., Squires J.E. (2018). Liver Transplantation for Propionic Acidemia and Methylmalonic Acidemia: Perioperative Management and Clinical Outcomes. Liver Transpl..

[218] Human Genomics in Global Health. https://www.who.int/genomics/public/geneticdiseases/en/index2.html#CF.

[219] Zuckerman J.B., McCoy K., Schechter M.S., Dorgan D., Jain M., MacDonald K.D., Callison C., Walker S., Bodie S., Barbier A. (2019). Safety and Tolerability of a Single Dose of MRT5005, a Inhaled CFTR mRNA Therapeutic, in Adult CF Patients (Poster 515). Pediatric Pulmonol..

[220] Waisbren S.E., Gropman A.L., Batshaw M.L., Members of the Urea Cycle Disorders Consortium (UCDC) (2016). Improving long term outcomes in urea cycle disorders-report from the Urea Cycle Disorders Consortium. J. Inherit. Metab. Dis..

[221] Brassier A., Gobin S., Arnoux J.B., Valayannopoulos V., Habarou F., Kossorotoff M., Servais A., Barbier V., Dubois S., Touati G. (2015). Long-term outcomes in Ornithine Transcarbamylase deficiency: A series of 90 patients. Orphanet J. Rare Dis..

[222] Intrado. https://www.globenewswire.com/news-release/2019/09/09/1913059/0/en/Translate-Bio-Announces-Pipeline-Program-Update.html.

[223] Shim G., Kim D., Park G.T., Jin H., Suh S.K., Oh Y.K. (2017). Therapeutic gene editing: Delivery and regulatory perspectives. Acta Pharmacol. Sin..

[224] Conway A., Mendel M., Kim K., McGovern K., Boyko A., Zhang L., Miller J.C., DeKelver R.C., Paschon D.E., Mui B.L. (2019). Non-viral Delivery of Zinc Finger Nuclease mRNA Enables Highly Efficient In Vivo Genome Editing of Multiple Therapeutic Gene Targets. Mol. Ther..

[225] Gersbach C.A., Gaj T., Barbas C.F. (2014). Synthetic zinc finger proteins: The advent of targeted gene regulation and genome modification technologies. Acc. Chem. Res..

[226] Yoshida K., Treen N. (2018). TALEN-Based Knockout System. Adv. Exp. Med. Biol..

[227] Wang H., Yang H., Shivalila C.S., Dawlaty M.M., Cheng A.W., Zhang F., Jaenisch R. (2013). One-step generation of mice carrying mutations in multiple genes by CRISPR/cas-mediated genome engineering. Cell.

[228] Zhang H.-X., Zhang Y., Yin H. (2019). Genome Editing with mRNA Encoding ZFN, TALEN, and Cas9. Mol. Ther..

[229] Crudele J.M., Chamberlain J.S. (2018). Cas9 immunity creates challenges for CRISPR gene editing therapies. Nat. Commun..

[230] Wang J., Exline C.M., DeClercq J.J., Llewellyn G.N., Hayward S.B., Li P.W.-L., Shivak D.A., Surosky R.T., Gregory P.D., Holmes M.C. (2015). Homology-driven genome editing in hematopoietic stem and progenitor cells using ZFN mRNA and AAV6 donors. Nat. Biotechnol..

[231] De Ravin S.S., Reik A., Liu P.-Q., Li L., Wu X., Su L., Raley C., Theobald N., Choi U., Song A.H. (2016). Targeted gene addition in human CD34+ hematopoietic cells for correction of X-linked chronic granulomatous disease. Nat. Biotechnol..

[232] Wang J., DeClercq J.J., Hayward S.B., Li P.W.-L., Shivak D.A., Gregory P.D., Lee G., Holmes M.C. (2015). Highly efficient homology-driven genome editing in human T cells by combining zinc-finger nuclease mRNA and AAV6 donor delivery. Nucleic Acids Res..

[233] Wefers B., Panda S.K., Ortiz O., Brandl C., Hensler S., Hansen J., Wurst W., Kühn R. (2013). Generation of targeted mouse mutants by embryo microinjection of TALEN mRNA. Nat. Protoc..

[234] Hwang W.Y., Fu Y., Reyon D., Maeder M.L., Tsai S.Q., Sander J.D., Peterson R.T., Yeh J.-R.J., Joung J.K. (2013). Efficient genome editing in zebrafish using a CRISPR-Cas system. Nat. Biotechnol..

[235] Watanabe M., Nakano K., Matsunari H., Matsuda T., Maehara M., Kanai T., Kobayashi M., Matsumura Y., Sakai R., Kuramoto M. (2013). Generation of Interleukin-2 Receptor Gamma Gene Knockout Pigs from Somatic Cells Genetically Modified by Zinc Finger Nuclease-Encoding mRNA. PLoS ONE.

[236] Liu J., Chang J., Jiang Y., Meng X., Sun T., Mao L., Xu Q., Wang M. (2019). Fast and Efficient CRISPR/Cas9 Genome Editing In Vivo Enabled by Bioreducible Lipid and Messenger RNA Nanoparticles. Adv. Mater..

[237] Samson M., Libert F., Doranz B.J., Rucker J., Liesnard C., Farber C.-M., Saragosti S., Lapoumeroulie C., Cognaux J., Forceille C. (1996). Resistance to HIV-1 infection in Caucasian individuals bearing mutant alleles of the CCR-5 chemokine receptor gene. Nature.

[238] Ware R.E., de Montalembert M., Tshilolo L., Abboud M.R. (2017). Sickle cell disease. Lancet.

[239] Smith E.C., Luc S., Croney D.M., Woodworth M.B., Greig L.C., Fujiwara Y., Nguyen M., Sher F., Macklis J.D., Bauer D.E. (2016). Strict in vivo specificity of the Bcl11a erythroid enhancer. Blood.

[240] Qasim W., Zhan H., Samarasinghe S., Adams S., Amrolia P., Stafford S., Butler K., Rivat C., Wright G., Somana K. (2017). Molecular remission of infant B-ALL after infusion of universal TALEN gene-edited CAR T cells. Sci. Transl. Med..

[241] Jo J.-I., Gao J.-Q., Tabata Y. (2019). Biomaterial-based delivery systems of nucleic acid for regenerative research and regenerative therapy. Regen. Ther..

[242] Jopling C., Boue S., Belmonte J.C.I. (2011). Dedifferentiation, transdifferentiation and reprogramming: Three routes to regeneration. Nat. Rev. Mol. Cell Biol..

[243] Eguizabal C., Carlos J., Belmonte I. (2013). Reprogramming: Future Directions in Regenerative Medicine. Semin. Reprod. Med..

[244] Yakubov E., Rechavi G., Rozenblatt S., Givol D. (2010). Reprogramming of human fibroblasts to pluripotent stem cells using mRNA of four transcription factors. Biochem. Biophys. Res. Commun..

[245] Takahashi K., Yamanaka S. (2006). Induction of Pluripotent Stem Cells from Mouse Embryonic and Adult Fibroblast Cultures by Defined Factors. Cell.

[246] Warren L., Manos P.D., Ahfeldt T., Loh Y.H., Li H., Lau F., Ebina W., Mandal P.K., Smith Z.D., Meissner A. (2010). Highly efficient reprogramming to pluripotency and directed differentiation of human cells with synthetic modified mRNA. Cell Stem Cell.

[247] Corritore E., Lee Y.-S., Pasquale V., Liberati D., Hsu M.-J., Lombard C.A., Van Der Smissen P., Vetere A., Bonner-Weir S., Piemonti L. (2016). V-Maf Musculoaponeurotic Fibrosarcoma Oncogene Homolog A Synthetic Modified mRNA Drives Reprogramming of Human Pancreatic Duct-Derived Cells into Insulin-Secreting Cells. Stem Cells Transl. Med..

[248] Orlando G., Gianello P., Salvatori M., Stratta R.J., Soker S., Ricordi C., Domínguez-Bendala J. (2014). Cell Replacement Strategies Aimed at Reconstitution of the β-Cell Compartment in Type 1 Diabetes. Diabetes.

[249] Koblas T., Leontovyc I., Loukotova S., Kosinova L., Saudek F. (2016). Reprogramming of Pancreatic Exocrine Cells AR42J Into Insulin-producing Cells Using mRNAs for Pdx1, Ngn3, and MafA Transcription Factors. Mol. Ther. Nucleic Acids.

[250] Elangovan S., Khorsand B., Do A.V., Hong L., Dewerth A., Komann M., Ross R.D., Sumner D.R., Allamargot C., Salem A.K. (2015). Chemically modified RNA activated matrices enhance bone regeneration. J. Control. Release.

[251] Badieyan Z.S., Berezhanskyy T., Utzinger M., Aneja M.K., Emrich D., Erben R., Schüler C., Altpeter P., Ferizi M., Hasenpusch G. (2016). Transcript-activated collagen matrix as sustained mRNA delivery system for bone regeneration. J. Control. Release.

[252] Park J.S., Suryaprakash S., Lao Y.H., Leong K.W. (2015). Engineering mesenchymal stem cells for regenerative medicine and drug delivery. Methods.

[253] Ryser M.F., Ugarte F., Thieme S., Bornhäuser M., Roesen-Wolff A., Brenner S. (2008). mRNA Transfection of CXCR4-GFP Fusion—Simply Generated by PCR—Results in Efficient Migration of Primary Human Mesenchymal Stem Cells. Tissue Eng. Part C Methods.

[254] Nowakowski A., Andrzejewska A., Boltze J., Nitzsche F., Cui L.L., Jolkkonen J., Walczak P., Lukomska B., Janowski M. (2017). Translation, but not transfection limits clinically relevant, exogenous mRNA based induction of alpha-4 integrin expression on human mesenchymal stem cells. Sci. Rep..

[255] Levy O., Zhao W., Mortensen L.J., Leblanc S., Tsang K., Fu M. (2013). mRNA-engineered mesenchymal stem cells for targeted delivery of interleukin-10 to sites of inflammation. Blood.

